# Furnace-Dependent Phase Evolution of Ball-Milled BaTiO_3_ Powders under Nominally Identical Calcination Conditions

**DOI:** 10.1021/acsomega.6c04998

**Published:** 2026-09-23

**Authors:** Mahmoud S. Alkathy, Person Pereira Neves, Aníbal Thiago Bezerra, Gabriel Monteiro A. da Rocha, Leandro Sanches, Jose Antonio Eiras

**Affiliations:** † Physics Department, 74347 Institute of Exact Sciences- UNIFAL-MG, Alfenas, Minas Gerais 37133-840, Brazil; ‡ Physics Department, Institute of Exact Sciences-Ufscar, São Carlos, São Paulo 13565-905, Brazil

## Abstract

In ceramic synthesis, the thermal history actually experienced by a powder may differ substantially from the programmed furnace schedule, with direct consequences for phase formation and reproducibility. This study shows that identical nominal calcination schedules can produce different BaTiO_3_ reaction outcomes when applied in different furnaces. A single high-energy ball-milled precursor batch was divided into three portions and calcined at 1200 °C for 8 h using the same programmed heating and cooling rates. XRD and Rietveld refinement identified tetragonal BaTiO_3_ (*P*4*mm*) as the dominant phase in all samples. However, the powder treated in Furnace 1 showed weak Ba-rich secondary phases, smaller crystallites, higher microstrain, and poorer refinement statistics, whereas samples from Furnaces 2 and 3 were phase-pure within the XRD detection limit and exhibited larger, less strained crystallites. Direct thermocouple measurements revealed the origin of these differences. Furnaces 2 and 3 closely maintained the programmed 1200 °C set point, while the sample position in Furnace 1 reached only approximately 1028 °C. SEM revealed a finer, more compact microstructure for Furnace 1 and coarser, more heterogeneous grains for Furnaces 2 and 3. EDS mapping confirmed homogeneous Ba, Ti, and O distributions in all powders, although quantitative EDS indicated a higher Ba/Ti ratio in the Furnace 1 sample. XPS showed comparable BaTiO_3_-type surface chemical environments and modest surface carbonation for all samples. These results establish furnace identity and actual temperature distribution as critical processing variables that should be monitored and explicitly reported in reproducibility-sensitive BaTiO_3_ and ceramic-synthesis studies.

## Graphical abstract

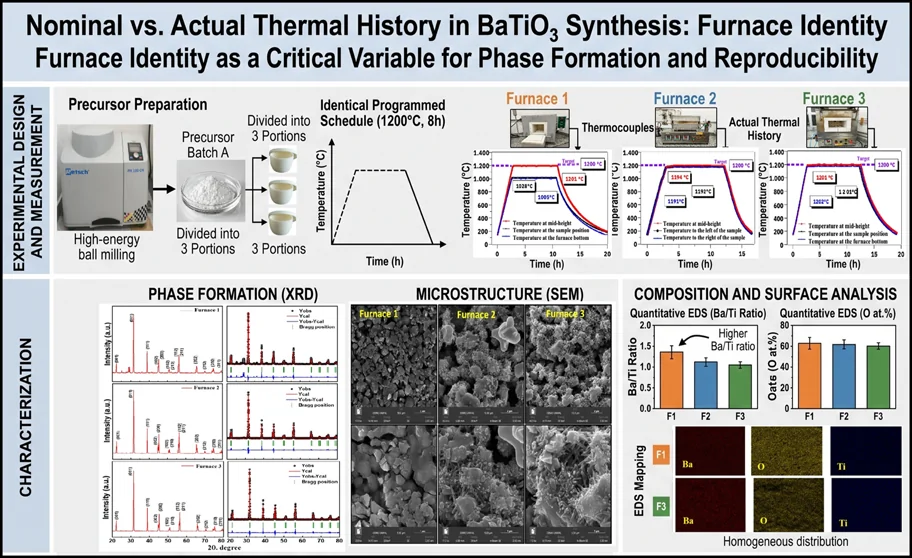

## 1 Introduction

Barium titanate (BaTiO_3_) is one of the most extensively studied ferroelectric perovskite oxides owing to its outstanding dielectric, ferroelectric, and piezoelectric properties. These characteristics have enabled its widespread application in multilayer ceramic capacitors, piezoelectric transducers, electro-optic devices, and other advanced electronic components.^1–4^ The functional performance of BaTiO_3_ is strongly dependent on its phase composition, crystal structure, grain size, and defect chemistry, making precise control of the synthesis process essential for obtaining materials with reproducible properties.^5,6^

Numerous studies have therefore focused on understanding the influence of processing parameters on the formation and evolution of BaTiO_3_. Calcination temperature, dwell time, heating and cooling rates, precursor chemistry, and milling conditions have all been shown to significantly affect phase formation, crystallinity, particle morphology, and dielectric performance.^7–10^ Among the available preparation methods, conventional solid-state synthesis remains attractive because of its simplicity, scalability, and low production cost. However, achieving complete reaction and phase purity often requires relatively high calcination temperatures and extended thermal treatments. Consequently, considerable effort has been devoted to optimizing thermal processing conditions.

In addition to conventional solid-state routes, several alternative synthesis techniques have been developed to produce BaTiO_3_ powders with improved homogeneity, reduced particle size, and lower processing temperatures. Solution-based methods, including sol-precipitation techniques, can generate chemically homogeneous precursors that facilitate phase formation at lower temperatures and yield finer powders compared with mechanically mixed oxide systems.^11^ Likewise, solvothermal, soft-chemistry, and RF plasma CVD approaches have demonstrated the ability to produce nanoscale tetragonal BaTiO_3_ with controlled morphology and high phase purity.^12–14^ Despite these advances, thermal treatment remains a critical step regardless of the synthesis route employed, as it ultimately governs crystallization, phase stability, grain growth, and defect evolution.

Although the effects of thermal schedules and processing atmospheres have been extensively investigated, the furnace itself is generally regarded as an ideal heat source and is rarely considered an independent processing variable. In most experimental studies, different furnaces are implicitly assumed to be interchangeable provided that the same programmed temperature, heating rate, cooling rate, and holding time are used.^15,16^ However, this assumption may not always be valid. Variations in thermocouple calibration, temperature distribution within the hot zone, heat-transfer characteristics, insulation design, air leakage, and gas-flow patterns can generate substantially different thermal environments, even when identical nominal operating conditions are selected.^17–19^ As a result, powders may experience different effective temperature histories, potentially influencing reaction kinetics, phase evolution, crystallite growth, and the formation of residual secondary phases.

The importance of this issue is amplified by the fact that accurate determination of the true temperature field inside a furnace remains challenging. Differences between programmed temperatures and actual local temperatures within the working zone are well documented, particularly when monitoring gas temperatures, furnace-wall temperatures, or spatial thermal gradients.^20–24^ Despite these challenges, the influence of furnace-specific thermal characteristics on the synthesis of ceramic materials has received relatively little attention. Consequently, furnace-to-furnace variability represents a potentially significant but often overlooked source of discrepancies between laboratories and may contribute to difficulties in reproducing published processing protocols.

To address this knowledge gap, the present study investigates whether furnace identity alone can influence the synthesis outcome of BaTiO_3_ powders under otherwise identical processing conditions. A single batch of high-energy ball-milled BaTiO_3_ precursor powder was calcined at 1200 °C for 8 h in air using three different industrially relevant furnaces operated with identical programmed heating and cooling schedules. Structural, microstructural, and compositional changes were systematically analyzed through Rietveld refinement of X-ray diffraction data, scanning electron microscopy coupled with EDS mapping, quantitative elemental analysis, and X-ray photoelectron spectroscopy (XPS); in addition, the temperature actually reached inside each furnace was directly verified using three-position thermocouple measurements, rather than assumed from the programmed set point alone. The results demonstrate that differences in furnace configuration and thermal environment are sufficient to modify phase assemblage, regulate the formation of Ba-rich intermediate phases, and influence crystallite size, microstrain, and particle morphology; direct thermocouple measurements further show that this behavior tracks a real, substantial deficit between the programmed and actually attained temperature in one of the three furnaces, while XPS confirms a consistent surface chemical environment across all three furnaces despite these differences. By identifying furnace identity and the actual thermal history it produces as a critical processing parameter, this work provides new insight into an often-neglected aspect of ceramic synthesis and offers practical guidance for improving interlaboratory reproducibility and the transferability of thermal processing protocols for BaTiO_3_ and related functional ceramics. Specifically, we test the hypothesis that furnace identity alone, under fixed nominal calcination conditions, can measurably affect phase assemblage, crystallite development, and microstructure in BaTiO_3_ powders. We emphasize that the aim of the present work is not to propose a new BaTiO_3_ synthesis route, which is a mature and extensively documented topic;^25,26^ rather, by holding the precursor, stoichiometry, milling, crucible, atmosphere and programmed thermal schedule fixed and varying only the furnace hardware, we test whether the common assumption of furnace interchangeability itself is valid, a factor that is rarely verified or even reported in the ceramic-processing literature.

## 2 Experimental Section

### 2.1 Materials Synthesized

BaTiO_3_ powder was synthesized via a conventional solid-state reaction route. High purity BaCO_3_ (99.99%) and TiO_2_ (≥99.8%) powders (Sigma-Aldrich) were used as starting materials. BaCO_3_ and TiO_2_ were weighed in a stoichiometric 1:1 molar ratio, using 2.5387 g of BaCO_3_ and 1.0275 g of TiO_2_ (total precursor batch mass 3.5662 g; molar ratio BaCO_3_:TiO_2_ = 1.000:1.000), prior to ball milling. The powders were thoroughly mixed and subjected to high energy ball milling for 4 h to obtain a homogeneous and finely dispersed precursor mixture. The milled powder was subsequently divided into three equal portions and placed in identical covered alumina crucibles. This experimental design was adopted to ensure that the furnace type constituted the sole intentional processing variable during calcination. Each portion was calcined in static laboratory air at 1200 °C for 8 h using the same thermal profile, consisting of a controlled heating rate of 5 °C min^–1^. The calcination treatments were carried out in three different laboratory furnaces: Furnace 1, a Trade Lab. Ambiental electric muffle furnace (model TLA200C, 3500 W, 16 A); Furnace 2, a FORTELAB FT-1300/H tube furnace (4000 W, 18 A); and Furnace 3, a FORNOS INTI 1300 ML/PCH Pechini muffle furnace (4000 W, 18 A). All three furnaces were operated in static laboratory air, with no forced or flowing gas atmosphere in any case, including Furnace 2, whose tube geometry refers only to the shape of its heating chamber and not to an active gas-flow system. In all cases, the samples were positioned at the center of the respective hot zones to minimize spatial temperature variations. The approximate dimensions of the heated chambers were 20 × 15 × 15 cm^3^ for Furnace 1, 28 cm × 14 cm (length × inner diameter) for the tube furnace (Furnace 2); and 25 × 23 × 12 cm^3^ for Furnace 3. Although identical nominal calcination conditions were employed, the distinct furnace geometries and heating configurations are expected to generate different thermal-field distributions and heat-transfer environments, potentially influencing the resulting microstructure and phase evolution of the BaTiO_3_ powders. To verify the temperatures actually attained inside each furnace, temperature profiles were recorded at three locations: at the midheight of the hot zone, at the sample (crucible) position, and near the furnace bottom. K-type thermocouples were used to continuously record the temperature throughout the thermal cycle, including the cooling stage. The resulting temperature profiles are presented and discussed in Section 3.1. Cooling in all three furnaces followed a programmed rate of 5 °C min^–1^ applied after the 8 h dwell; owing to the thermal inertia of the furnaces, the actual cooling behavior did not strictly follow this programmed ramp, and the effective cooling history experienced by each furnace corresponds directly to the temperature profiles shown in Figure 1. All three portions were calcined in separate runs, one in each furnace, using the same thermal program, with each furnace sampled once under nominally identical laboratory conditions.

**Figure 1:**
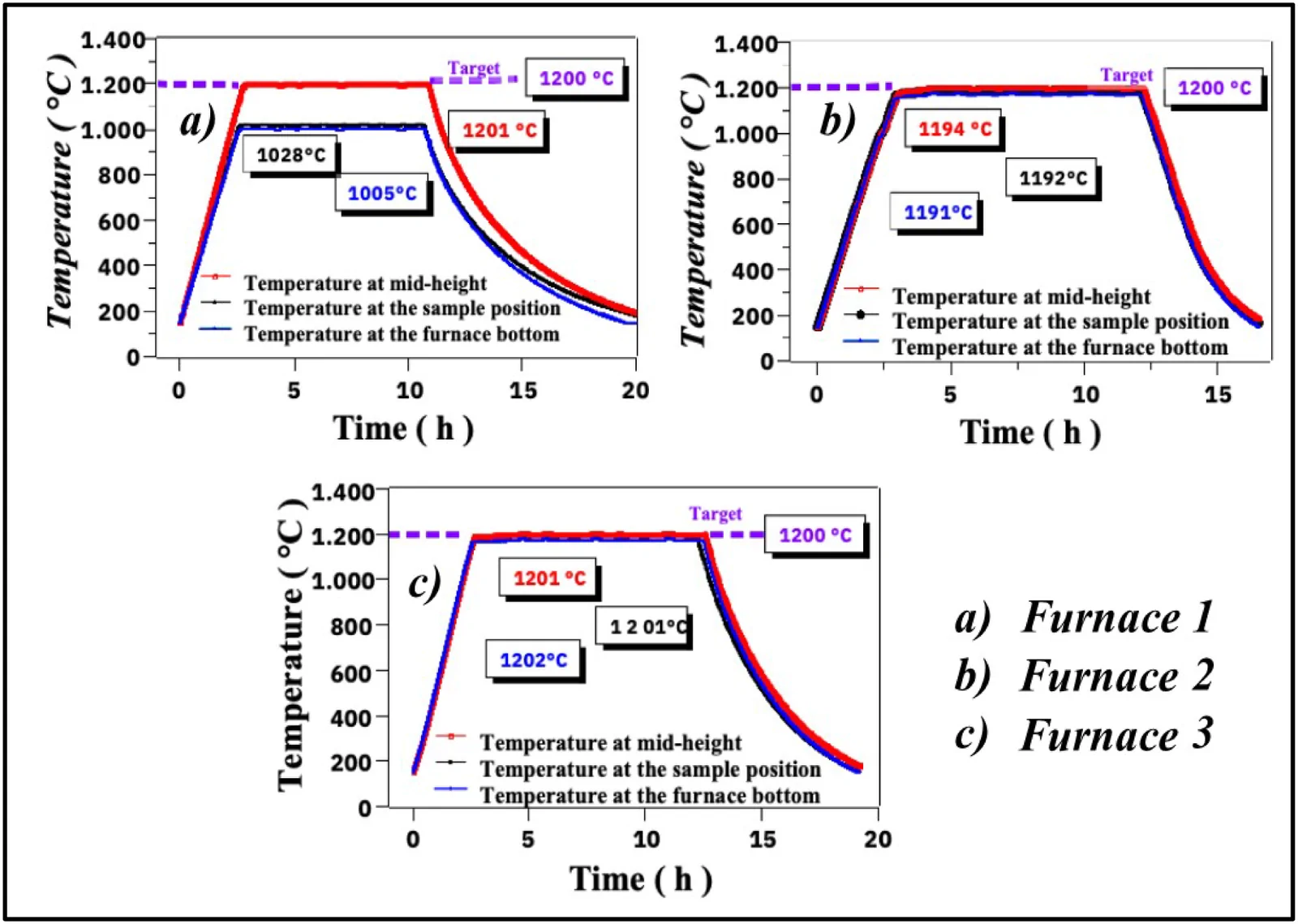
Temperature profiles measured by three K-type thermocouples (midheight, sample position, and furnace bottom) during the programmed 1200 °C/8 h calcination schedule in (a) Furnace 1, (b) Furnace 2, and (c) Furnace 3. Furnaces 2 and 3 are essentially isothermal and closely track the 1200 °C set point at all three positions (1191–1194 °C and 1201–1202 °C, respectively), whereas Furnace 1 shows a substantial spatial temperature deficit: the sample position reaches only 1028 °C and the furnace bottom only 1005 °C, roughly 170–195 °C below both the midheight reading (1201 °C) and the nominal target.

### 2.2 Materials Characterization

After calcination, the powders were lightly crushed and analyzed using X-ray diffraction (XRD). Diffraction patterns were recorded using Cu Kα radiation (λ = 1.542 Å) on a Rigaku Ultima IV diffractometer over a 2θ range of 20–80°. The step size was 0.02°, and the counting duration was 3 s per step. The FullProf Suite (version 5.20) was used to refine structural parameters using the Rietveld method, with a tetragonal BaTiO_3_ structure assumed (space group *P*4*mm*). Scanning electron microscopy (SEM) was used to examine the microstructure, morphology, and elemental distribution of the calcined powders. The samples were crushed and coated with a 10 nm gold layer using a sputter coater (Denton Vacuum, Desk V). A TESCAN MIRA4 FEG microscope equipped with Everhart–Thornley secondary electron (SE), in-beam SE and low-energy backscattered electron (BSE) detectors was used for imaging at accelerating voltages ranging from 15 to 20 kV. Elemental spectra and compositional maps were obtained using energy-dispersive X-ray spectroscopy (EDS) with an SDD detector (Ultim Max 100, Oxford Instruments). EDS experiments were carried out at accelerating voltages of 15–20 kV. All microscopy analyses were performed at the Centro de Microscopia, Universidade Federal de Alfenas, Minas Gerais, Brazil. For each powder, quantitative SEM–EDS analyses were performed on multiple regions, and the scatter of the measured elemental fractions is consistent with the typical uncertainty range (a few atomic percent) reported for SEM–EDS measurements of oxide ceramics. X-ray photoelectron spectroscopy (XPS) survey and high-resolution spectra (Ba 3d, Ti 2p, O 1s, and C 1s regions) were additionally acquired for the calcined powders from all three furnaces using a Scienta Omicron X-ray photoelectron spectrometer (MATRIX V3.2 acquisition software) with a monochromatic Al Kα X-ray source (1486.7 eV, 15 kV anode voltage), operated in fixed analyzer transmission (FAT) mode at normal (0°) emission angle to provide a surface-sensitive, independent check on their chemical states and compositions; for each powder, the binding-energy scale was referenced to the adventitious C 1s (C–C/C–H) peak fixed at 284.8 eV.

## 3 Results and Discussion

### 3.1 Furnace Temperature Verification

Because furnace geometry and heat-transfer characteristics can, in principle, produce different effective thermal histories even under identical programmed schedules, the temperature actually reached inside each furnace was verified directly using three K-type thermocouples positioned at the midheight of the hot zone, at the sample (crucible) position, and at the furnace bottom (Section 2, Figure 1). Furnace 2 and Furnace 3 are essentially isothermal across all three monitored positions and closely track the programmed 1200 °C set point (Furnace 2:1194 °C, 1191 °C, and 1192 °C at midheight, sample position, and furnace bottom, respectively; Furnace 3:1201 °C, 1202 °C, and 1201 °C). Furnace 1, by contrast, shows a substantial spatial temperature deficit: the midheight reading (1201 °C) reaches the programmed set point, but the sample itself experienced only 1028 °C, with the furnace bottom reading lower still at 1005 °C, a shortfall of roughly 170–195 °C relative to both the midheight reading and the nominal target. Because all three furnaces were operated in static laboratory air with no forced or flowing gas atmosphere, furnace-to-furnace differences in gas circulation can be excluded as a contributing factor. This measured temperature deficit provides a direct, quantitative basis for interpreting the furnace-dependent differences in phase assemblage, crystallite size, and microstrain discussed in the following sections.

### 3.2 XRD Analysis

Figures 2, 3, 4 show Rietveld-refined XRD patterns of BaTiO_3_ powders calcined at 1200 °C for 8 h in Furnace 1, Furnace 2, and Furnace 3. In all three samples, the major reflections align with the perovskite phase BaTiO_3_, with peak positions closely matching the tetragonal BaTiO_3_ phase (space group *P*4*mm*) reported in standard diffraction data and structural studies.^27–29^ However, closer inspection of the diffraction profiles reveals minor but systematic differences between the furnaces. The powders from Furnace 2 and Furnace 3 exhibit sharp, well-defined and symmetric reflections over the investigated 2θ range. The observed (Y_obs_) and calculated (*Y*_cal_) profiles are nearly identical, and the difference curves (*Y*_obs_ – *Y*_cal_) fluctuate about zero with only minor random variations, indicating an excellent fit without significant modeled phases. The refined a-parameter ranges from 3.993 to 3.998 Å, while the c-parameter ranges from 4.025 to 4.033 Å, yielding c/a ratios of approximately 1.0068, 1.0091, and 1.0100 for powders from Furnace 1, Furnace 2, and Furnace 3, respectively. These small variations indicate that the tetragonal distortion of BaTiO_3_ is largely preserved regardless of the furnace employed, and the unit-cell volumes, which cluster around 64.3 Å^3^, confirm that the average crystal structure remains essentially unchanged within experimental uncertainty.^30^ In contrast, the Furnace-1 pattern shows weak additional peaks superimposed on one of the major BaTiO_3_ reflections, accompanied by noticeably higher refinement residuals. The Furnace-1 powder has the highest *R*_wp_ (≈20.3%) and χ^2^ (≈3.28), whereas the Furnace-2 and Furnace-3 samples exhibit much lower *R*_wp_ values (≈12.0 and 11.6%) and χ^2^ (≈2.31 and 2.03), with correspondingly lower *R*_p_ and *R*_ex_ values; this trend indicates that the calculated profiles reproduce the experimental data more faithfully for Furnaces 2 and 3 than for Furnace 1. The extra peaks in the Furnace-1 sample can be attributed to Ba-rich intermediate oxides, such as BaTi_2_O_5_, which are known Ba–Ti–O phases that may persist if the solid-state reaction is not fully driven to completion.^31^ The presence of these minor reflections, together with the inferior refinement statistics, strongly suggests a genuine crystalline secondary phase rather than a microstructural peak-shape artifact.^31^ We note that incompletely reacted BaCO_3_ and other intermediate barium titanate phases have also been reported to persist during BaTiO_3_ formation from BaCO_3_ and TiO_2_,^32^ while the elevated bulk Ba/Ti ratio measured by EDS for the Furnace-1 sample (Section 3.5) is consistent with a Ba-rich phase, we cannot fully exclude a contribution from residual, incompletely reacted carbonate on the basis of the present laboratory XRD data alone, and a definitive phase assignment would require comparison against reference diffraction patterns at higher angular resolution. Complementary XPS analysis of the calcined powders (Section 3.6) shows a carbonate-type carbon component present in all three furnaces, which is consistent with, though not uniquely diagnostic of, a residual-carbonate or Ba-rich secondary phase specifically in Furnace 1. To test the phase identity of the unindexed reflections directly, we simulated the powder XRD pattern of BaTi_2_O_5_ (monoclinic, space group C2, a = 16.899(3) Å, b = 3.9350(6) Å, c = 9.4105(15) Å, β = 103.103(3)° from its published single-crystal structure,^33^ using kinematic structure-factor calculations for Cu Kα radiation, and included the resulting Bragg-position tick marks alongside those of tetragonal BaTiO_3_ in Figure 2. All 14 previously unindexed reflections, including the two most intense ones at 28.82° and 29.34°, coincide with predicted BaTi_2_O_5_ Bragg positions to within 0.01–0.30°, whereas equivalent comparisons against BaCO_3_ and anatase TiO_2_ (tested using the same approach) show much larger, inconsistent deviations of up to 0.9° and are not supported. We therefore identify BaTi_2_O_5_ as the most probable secondary phase in the Furnace-1 powder. We note that this comparison uses calculated relative intensities from the reported crystal structure rather than a full multiphase Rietveld corefinement; incorporating BaTi_2_O_5_ explicitly into the refinement, allowing joint fitting of scale factors, backgrounds and peak shapes for both phases, would provide a quantitative phase fraction and is a natural extension of the present analysis. Considering the counting statistics, instrumental background and peak overlaps in our measurements, the practical detection limit of the laboratory XRD configuration used here is on the order of 1–3 wt % for crystalline secondary phases in typical laboratory setups similar to ours. Therefore, the Ba-rich intermediate oxides present in the Furnace-1 powder are expected to be confined to trace amounts within this range, which is consistent with their very low peak intensities and the nearly unchanged average BaTiO_3_ lattice parameters.

**Figure 2:**
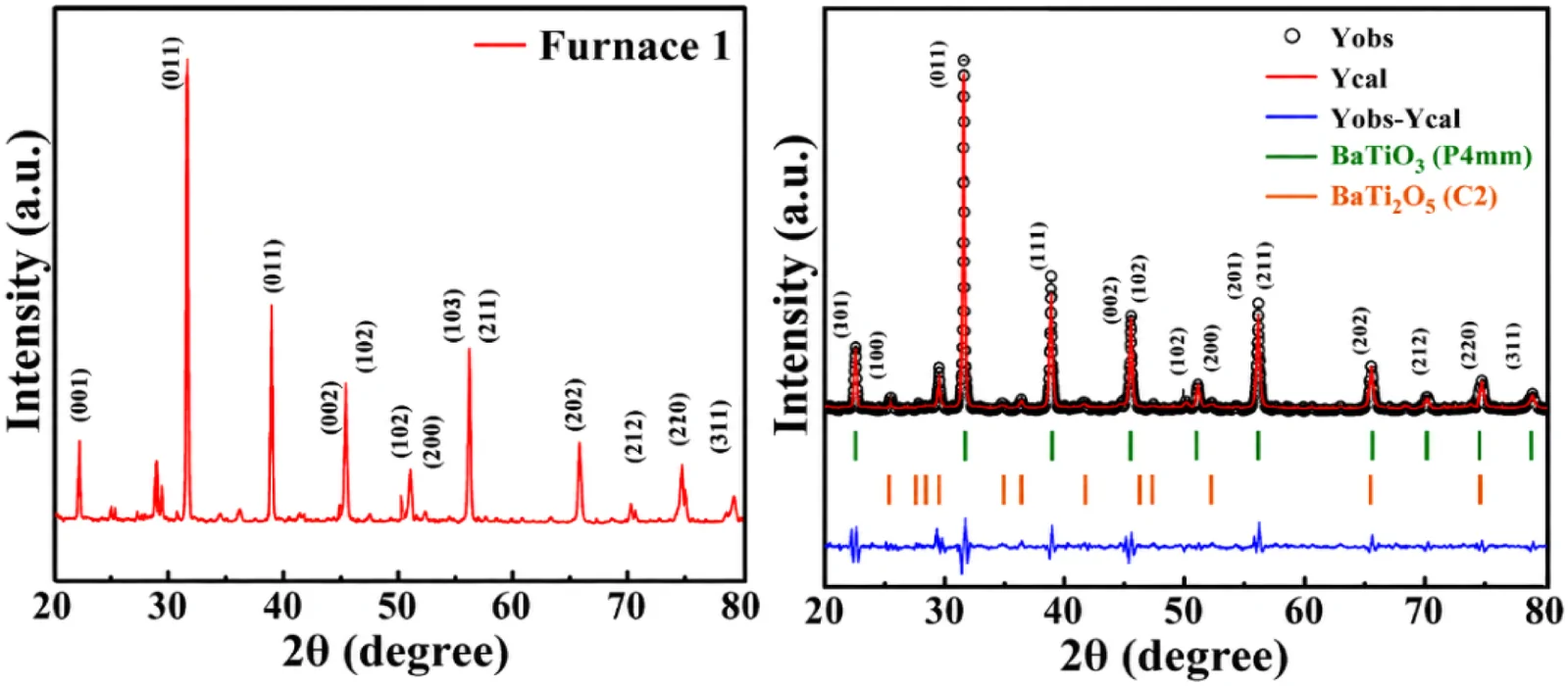
X-ray diffraction pattern and two-phase Rietveld refinement of BaTiO_3_ powder calcined at 1200 °C for 8 h in Furnace 1, showing the tetragonal BaTiO_3_ (*P*4*mm*) and monoclinic BaTi_2_O_5_ (C2) phases respectively, together with the observed, calculated, and difference profiles, Bragg peak positions for both phases, and the corresponding refinement residuals.

**Figure 3:**
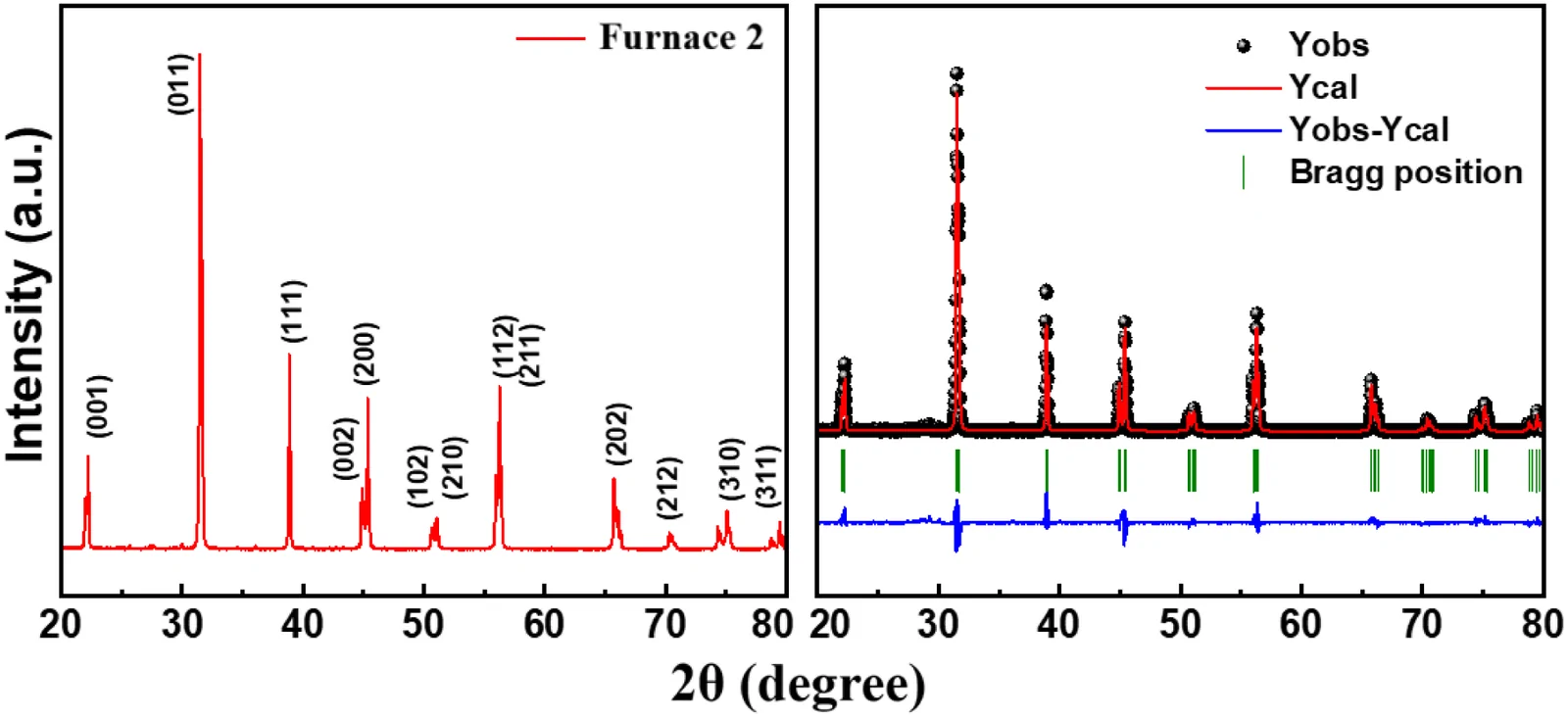
X-ray diffraction pattern and Rietveld refinement of BaTiO_3_ powder calcined at 1200 °C for 8 h in Furnace 2, confirming phase-pure tetragonal BaTiO_3_ with good agreement between observed and calculated profiles and low refinement residuals.

**Figure 4:**
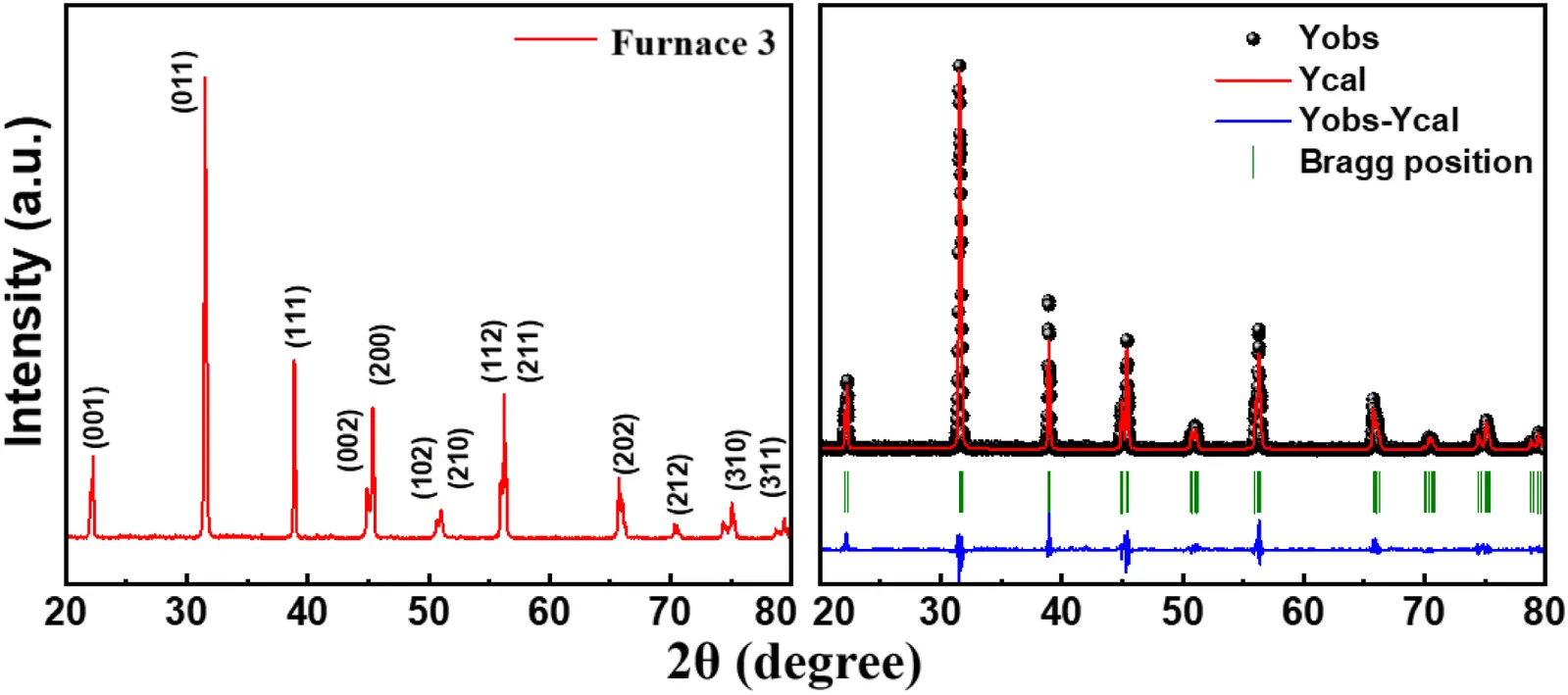
X-ray diffraction pattern and Rietveld refinement of BaTiO_3_ powder calcined at 1200 °C for 8 h in Furnace 3, showing phase-pure tetragonal BaTiO_3_ with sharp peaks, low residuals and the largest average crystallite size among the three furnaces.

The crystallite size and microstrain were estimated using parameters obtained from Gaussian-fitted profiles of the (011) diffraction peak as shown in Figure 5a, applied within the Scherrer equation. Two representative diffraction peaks were selected and fitted with Gaussian functions to determine the corrected full width at half-maximum (*fwhm*), denoted as β_1_ and β_2_, at their respective Bragg angles θ_1_ and θ_2_. The corresponding crystallite sizes (*D*_1_ and *D*_2_) were then calculated using the Scherrer equation:^34^

1 $$D_{1}=\frac{K\lambda }{\beta_{1}\cos\theta_{1}}$$

2 $$D_{2}=\frac{K\lambda }{\beta_{2}\cos\theta_{2}}$$

Then the average crystallite size was taken as

3 $$D_{\mathrm{avg}}=\frac{D_{1}+D_{2}}{2}$$

The lattice strain associated with each peak was estimated using a formula:^35^

4 $$\varepsilon_{1}=\frac{\beta_{1}}{4\tan\theta_{1}}$$

5 $$\varepsilon_{2}=\frac{\beta_{2}}{4\tan\theta_{2}}$$

And the average microstrain was obtained using

6 $$\varepsilon_{\mathrm{avg}}=\frac{\varepsilon_{1}+\varepsilon_{2}}{2}$$

Table 1 shows that the crystallite size for Furnace 1 powder is approximately 53 nm, while samples from Furnace 2 and Furnace 3 had greater average crystallite sizes of 78 and 81 nm, respectively. The average crystallite sizes initially estimated from Scherrer analysis of the (101) and (011) peaks are approximately 53, 78, and 81 nm for powders from Furnace 1, Furnace 2 and Furnace 3, respectively (Table 1).

**Figure 5:**
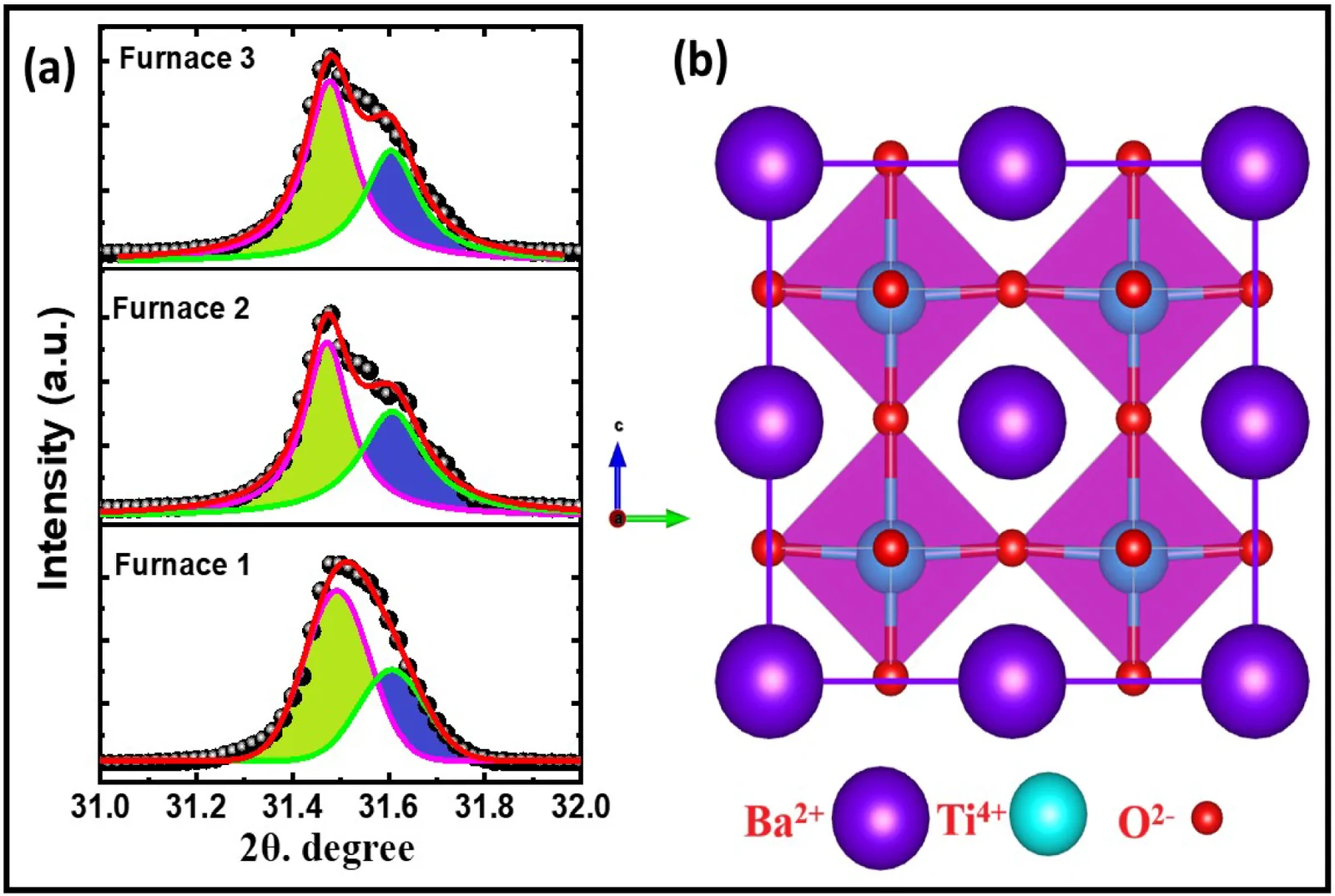
(a) Gaussian fitting of the (011) BaTiO_3_ reflection for powders calcined in Furnace 1, Furnace 2, and Furnace 3 (bottom to top), used to extract crystallite size and lattice strain. (b) Schematic diagram of the tetragonal BaTiO_3_ structure, showing Ba at the corners, Ti at the center, and oxygen atoms forming the distorted TiO_6_ octahedra.

**Figure 6:**
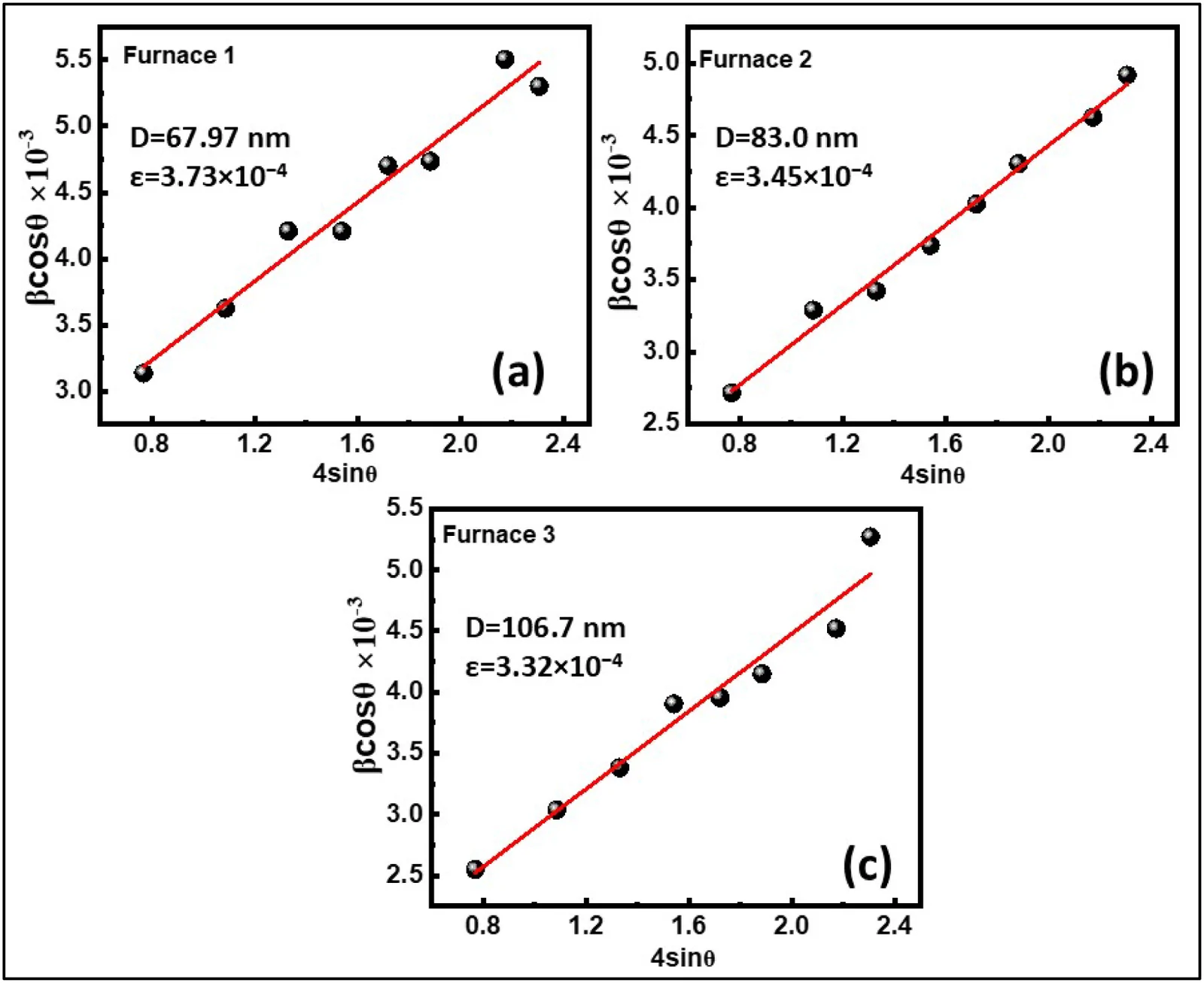
Williamson–Hall plots of *β* cos *θ* versus 4 sin *θ* for BaTiO_3_ powders calcined in (a) Furnace 1, (b) Furnace 2 and (c) Furnace 3, with linear fits used to extract crystallite size and lattice microstrain.

**Table 1:** Rietveld Refinement Parameters of Tetragonal Barium Titanate, Including Lattice Constants, Reliability Factors, and Structural Parameters Confirming the Formation of the BaTiO_3_ Calcined Powder at Three Different Furnaces

Parameter	Furnace 1	Furnace 2	Furnace 3
Space group	*P*4*mm*	*P*4*mm*	*P*4*mm*
a = b (Å)	3.9980	3.9944	3.9932
c (Å)	4.025	4.0304	4.0330
c/a	1.006753	1.009013	1.009967
V (Å^3^)	64.3499	64.3080	64.3087
*R*_wp_ (%)	20.3	12.0	11.6
*R*_p_ (%)	14.0	10.9	10.8
*R*_ex_ (%)	11.2	7.88	7.14
χ^2^	3.28	2.31	2.03
β_1_ (deg)	0.1327 ± 0.016	0.10735 ± 0.009	0.1045 ± 0.014
β_2_ (deg)	0.1405 ± 0.025	0.1070 ± 0.0148	0.1028 ± 0.019
2θ_1_ (deg)	31.49 ± 0.016	31.47 ± 0.0012	31.44 ± 0.011
2θ_2_ (deg)	31.60 ± 0.033	31.60 ± 0.0021	31.54 ± 0.001
D_1_ (nm)	64.99	80.6	82.52
D_2_ (nm)	61.4	80.63	83.91
D _average_ (nm)	63.195	80.61	83.21
D (nm) from H–W	67.97	83.0	106.70
Microstrain × 10^–4^	3.73	3.45	3.32

#### 3.2.1 Williamson–Hall Analysis

The crystallite size and lattice microstrain of the BaTiO_3_ powders were also quantified using the Williamson–Hall (*W–H*) approach within the uniform deformation model. In this framework, the size- and strain-induced contributions to the X-ray peak broadening are separated through the linear relation:^36^

$$\beta \cos\theta =\frac{k\lambda }{D}+4\varepsilon \sin\theta$$

in which *β* is the full width at half-maximum (fwhm) of the diffraction peak, *θ* is the Bragg angle, *K* is the shape factor (taken as 0.9), *λ* is the Cu Kα wavelength (1.5406 Å), *D* is the volume-averaged crystallite size and *ε* denotes the lattice microstrain. Plots of *β* cos *θ* as a function of 4 sin *θ* for the powders calcined in the three furnaces are well described by straight lines, indicating that the *W–H* model provides an adequate description for these samples. From the slopes and intercepts of these fits shown in Figure 6a–c, the crystallite sizes are estimated to be about 67.97, 83, and 106.7 nm for powders from Furnace 1, Furnace 2 and Furnace 3, respectively, with corresponding microstrain values of 3.73 × 10^–4^, 3.45 × 10^–4^ and 3.32 × 10^–4^. These findings are consistent with the simpler Scherrer analysis and demonstrate that Furnace 1 creates significantly finer, more highly strained crystallites, whereas Furnaces 2 and 3 promote more widespread grain development with a minor reduction in average microstrain. The larger crystallite sizes obtained from the Williamson–Hall analysis relative to the simple Scherrer estimates are consistent with the partial separation of strain and size broadening reported in previous BaTiO_3_ and oxide nanoparticle studies. Previous line-profile investigations of oxide nanomaterials have shown that structural rearrangement and uniform grain development reduce strain-related peak broadening, resulting in increased crystallite size and decreased microstrain.^37^

The furnace-dependent differences in phase assemblage, crystallite size and microstrain in the BaTiO_3_ powders can be rationalized using basic thermodynamic and kinetic arguments. All samples were produced from the same ball-milled precursor and were nominally calcined under identical conditions in air (temperature, dwell time and heating rate), yet their microstructures are clearly distinct, indicating that the effective thermal environment in each furnace is a critical processing variable. Grain growth in BaTiO_3_ is driven by the reduction of grain-boundary and elastic strain energies and follows curvature-driven kinetics; because grain-boundary mobility and cation diffusivity exhibit Arrhenius-type temperature dependences, relatively small variations in the actual temperature field are sufficient to alter coarsening rates and reaction pathways.^38,39^ Furnace-specific factors such as offsets in peak temperature and temperature gradients within the hot zone can therefore modify diffusion kinetics and the progression of the solid-state reaction; indeed, the direct thermocouple measurements described in Section 2 show that this is precisely the case for Furnace 1, where the sample position reached only ∼1028 °C against a programmed set point of 1200 °C.^38,39^ Within this framework, the finer crystallite size and higher microstrain of the powder from Furnace 1 are naturally associated with a less favorable thermal profile that limits grain-boundary mobility and slows strain relaxation, so that Ba-rich intermediates are not fully consumed and a metastable microstructure is retained, in line with established BaO–TiO_2_ phase equilibria and BaTiO_3_ formation studies.^38–40^ By contrast, the larger crystallites, lower microstrain and absence of detectable secondary phases in the powders from Furnaces 2 and 3 point to more uniform and slightly more energetic thermal histories that enhance diffusion and drive the reaction closer to equilibrium, yielding phase-pure BaTiO_3_ with a more relaxed lattice.^40–42^ This interpretation is consistent with the defect chemistry of BaTiO_3_, in which local oxygen partial pressure and thermal history control the concentration and mobility of oxygen vacancies, thereby influencing cation diffusion and the stability of intermediate Ba–Ti–O phases.^43–45^ In this way, furnace-specific thermal fields become directly coupled to phase evolution, crystallite growth and strain relaxation during calcination.

Taken together, these results demonstrate that furnace-dependent effects can substantially modify the phase composition and microstructural state of BaTiO_3_, even when the nominal processing parameters are held constant. Furnace identity should thus be treated as an explicit processing parameter when designing, optimizing and transferring synthesis protocols, in line with observations for other perovskite systems where subtle differences in thermal treatment have been shown to govern structural evolution and functional performance.^41,42,46^ Related ideas of pathway-dependent phase evolution have also been discussed in molecular self-assembly systems, where precisely designed building blocks generate well-defined intermediate states that guide structure formation; we mention these works only as a broader conceptual analogy, to highlight that controlling intermediate states is a general route to regulating phase evolution, even though the chemistries and length scales are very different from the solid-state BaTiO_3_ reactions studied here.^47,48^

### 3.3 Morphological Analysis

SEM micrographs acquired at two magnifications (Figure 7) provide clear evidence of furnace-dependent microstructural evolution in the calcined powders. The BaTiO_3_ powder processed in Furnace 1 predominantly consists of compact, equiaxed grains with relatively smooth surfaces, indicative of limited grain coarsening, which is consistent with reports of BaTiO_3_ obtained by conventional solid-state routes near 1200–1300 °C.^40–42,46^ The morphology is consistent with the smaller crystallite size and higher microstrain obtained from the XRD/Williamson–Hall analysis, as well as with the persistence of faint Ba_2_TiO_4_/BaTi_2_O_5_-type reflections that signal an incomplete reaction in the BaO–TiO_2_ system. In contrast, the powders produced in Furnace 2 and Furnace 3 exhibit progressively coarser and more heterogeneous morphologies, characterized by small particles decorating larger grains and, in the case of Furnace 3, by a high density of acicular or whisker-like crystallites growing from the surfaces of coarser particles. These elongated features are typically associated with anisotropic growth driven by transient intermediate phases or local supersaturation at elevated effective temperatures, and they agree well with the larger crystallite sizes and lower microstrain values derived from Rietveld/Williamson–Hall analysis and with the disappearance of Ba-rich intermediate phases.^38,39,49,50^ In the BaO–TiO_2_ system, Ba-rich intermediates such as BaTi_2_O_5_ can form and then react to BaTiO_3_ at high temperature, creating transient liquid-like interfacial layers and locally supersaturated regions that favor directional growth along specific crystallographic directions.^38,39,49–51^ In our case, the lower effective calcination temperature experienced by the powder in Furnace 1 relative to the nominal target is likely to limit these effects, favoring equiaxed particles and a less advanced reaction, whereas Furnaces 2 and 3, which more closely reach the intended 1200 °C set point, promote a more complete reaction and enhanced grain growth, including epitaxial, whisker-like growth of BaTiO_3_ from the surfaces of coarser grains in Furnace 3. The direct three-position thermocouple measurements described in Section 2 (Figure 1) show that Furnaces 2 and 3 are both close to isothermal across the monitored positions and closely track the 1200 °C set point (Furnace 2:1191–1194 °C; Furnace 3:1201–1202 °C), whereas Furnace 1 shows a substantial temperature deficit specifically at the sample position (∼1028 °C, roughly 170 °C below target). This quantitatively accounts for the finer, more strained, incompletely reacted microstructure and residual Ba-rich phase observed in Furnace 1, which was effectively calcined at a much lower temperature than nominally programmed. The measurements do not, however, explain why Furnace 2 and Furnace 3, both operated at essentially the same near-target temperature, nevertheless differ from each other in the degree of whisker-like growth (more pronounced in Furnace 3 than in Furnace 2); resolving that remaining difference would require additional furnace-specific, spatially resolved measurements (e.g., of local heating/cooling rate or minor humidity differences in the ambient air), which are beyond the scope of the present comparison. Overall, the SEM observations corroborate the diffraction results, showing that Furnace 1 yields a finer, more strained and less fully reacted microstructure, whereas Furnaces 2 and 3 promote more extensive grain growth and the formation of phase-pure BaTiO_3_ with a more relaxed microstructure, in line with previous phase-equilibria and nonstoichiometry studies on BaTiO_3_ and related Ba-rich titanates.

**Figure 7:**
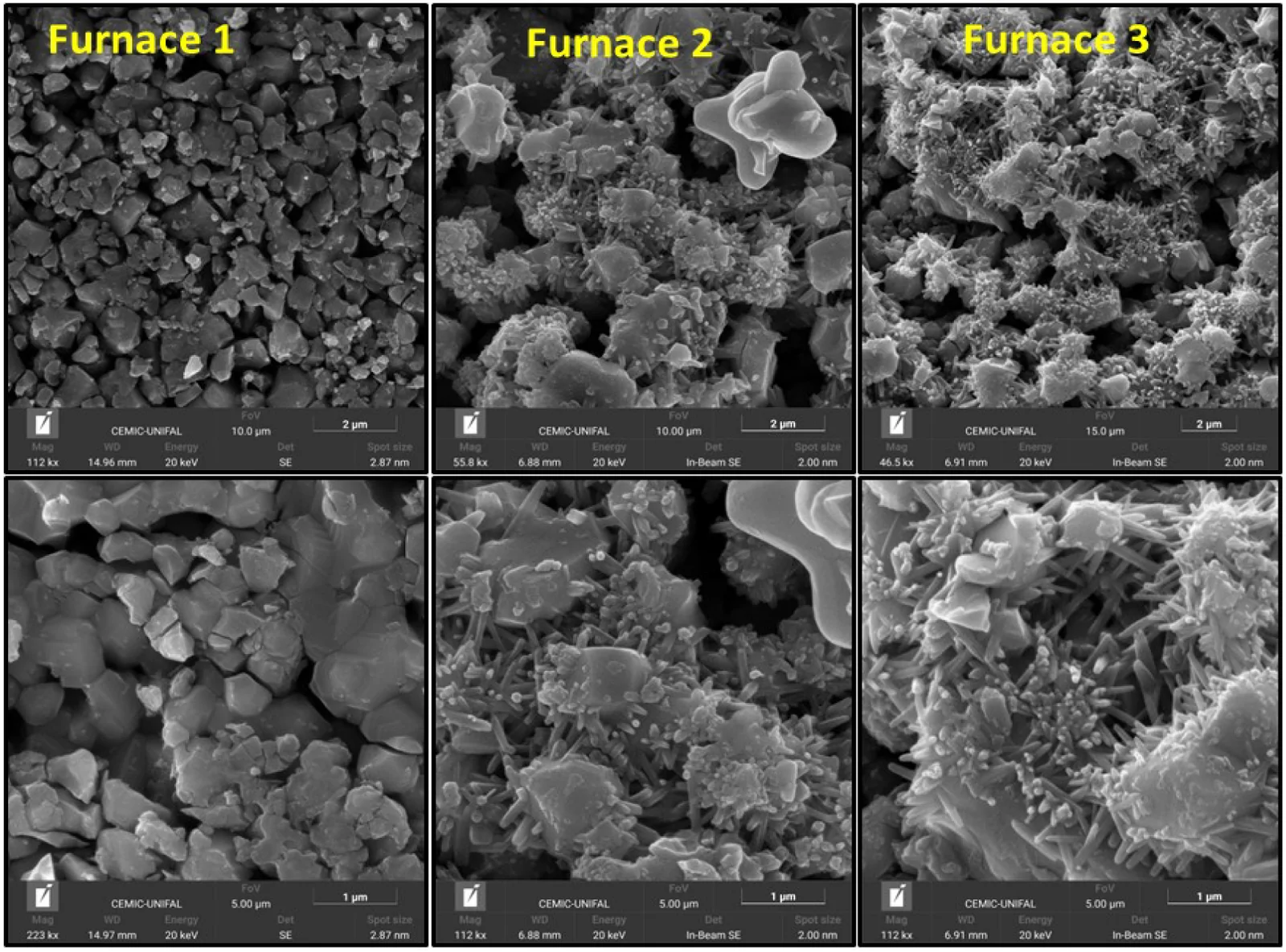
SEM micrographs of BaTiO_3_ powders calcined at 1200 °C for 8 h in Furnace 1, Furnace 2 and Furnace 3 at two magnifications.

The crystal structure and morphology obtained here are broadly consistent with previous reports of BaTiO_3_ prepared by conventional solid-state reaction at 1200–1300 °C, which typically show phase-pure tetragonal BaTiO_3_ with equiaxed submicrometric grains after prolonged calcination.^41,42,46,49^ However, most studies using similar calcination temperatures report relatively compact or slightly agglomerated particle morphologies, and do not describe the pronounced whisker-like features observed in the powders processed in Furnaces 2 and 3. This suggests that, under otherwise comparable processing conditions (starting reagents, milling time, calcination temperature and dwell time), the furnace configuration and associated thermal field (e.g., temperature gradients and heat-transfer mode) play a decisive role in promoting anisotropic BaTiO_3_ grain growth in our case, as anticipated by the sensitivity of BaTiO_3_ phase equilibria and defect chemistry to thermal conditions. In particular, the electric muffle furnace (Furnace 1) yields a more conventional equiaxed morphology, whereas the tube and Pechini-type muffles (Furnaces 2 and 3) favor the development of acicular crystallites, indicating that the furnace type can be a critical, yet often overlooked, processing parameter in solid-state synthesis of BaTiO_3_

### 3.4 EDS-Mapping Analysis

Figure 8 displays sample EDS elemental maps for Ba, O, and Ti obtained from the calcined powders of Furnace 1, Furnace 2, and Furnace 3. In all instances, the three elements are uniformly distributed across the examined regions, exhibiting no discernible segregation or compositional zoning at the micrometer scale, which is consistent with the formation of a single BaTiO_3_-based phase within the BaO–TiO_2_ system.^38,39^ The Ba and Ti maps exhibit a constant speckled contrast that closely aligns with the underlying particle shape, whereas the O signal encompasses the whole field of view, indicative of the creation of a singular Ba–Ti–O phase in line with the reported BaTiO_3_ homogeneity range and limited BaO solubility.^38,39,43^ No Ba-rich or Ti-rich clusters attributable to residual intermediate oxides are detected within the spatial resolution of the mapping, which is compatible with near-stoichiometric BaTiO_3_ compositions where nonstoichiometric deviations remain small.^43–45^ Collectively, our findings suggest that, at the microscale examined by SEM–EDS, all powders are chemically similar to the nominal BaTiO_3_ composition, in agreement with prior studies on nonstoichiometry and defect chemistry in undoped BaTiO_3_.^44,45^ Any residual BaTi_2_O_5_-type secondary domains in the Furnace-1 sample must consequently be either present in quantities below the detection threshold of EDS mapping or so finely scattered that they do not manifest as discrete compositionally segregated regions, as expected for Ba-rich equilibrium phases bordering the BaTiO_3_ field in the BaO–TiO_2_ phase diagram.^38,39^

**Figure 8:**
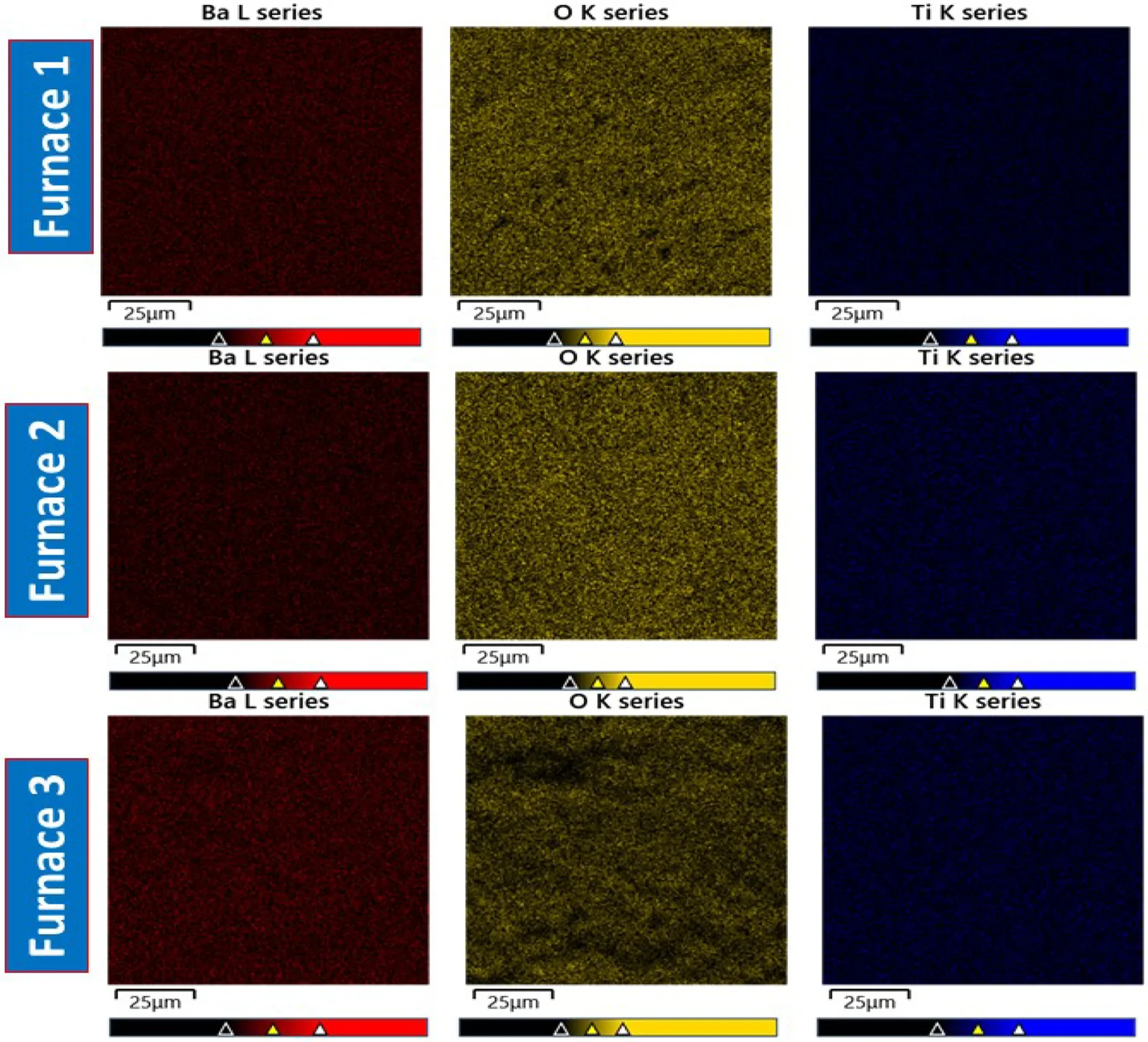
EDS elemental maps of Ba (Ba L series), O (O K series) and Ti (Ti K series) for BaTiO_3_ powders calcined at 1200 °C for 8 h in Furnace 1, Furnace 2 and Furnace 3. The Ba, Ti and O signals are homogeneously distributed over the analyzed regions for all three samples, with no micron-scale elemental segregation or foreign inclusions, indicating that the powders form a chemically uniform Ba–Ti–O matrix at the mapping scale.

### 3.5 EDS Quantitative Analyses

Figure 9 displays representative EDS quantitative spectra for the BaTiO_3_ powders calcined in the three different furnaces. The corresponding statistical parameters of the atomic fractions (maximum, minimum, average and standard deviation) are summarized in Table 2 and reveal systematic compositional differences induced by the furnace conditions. For ideal stoichiometric BaTiO_3_, the theoretical atomic percentages are 20 at. % Ba, 20 at. % Ti and 60 at. % O. Relative to these nominal values, the powders produced in Furnace 2 and Furnace 3 are markedly closer to the expected composition than the powder obtained in Furnace 1. In our measurements, the scatter of the elemental fractions over multiple analysis points corresponds to uncertainties of roughly ±0.5–1.0 at. % for Ba and Ti, i.e., relative errors on the order of 3–5%. These values are consistent with commonly reported error ranges for SEM–EDS quantification in oxide ceramics under similar acquisition conditions.^43–45,52^ Within this uncertainty window, the Ba/Ti ratios obtained for the powders from Furnaces 2 and 3 can be regarded as essentially stoichiometric.^43–45,52^ The powder calcined in Furnace 2 exhibits average atomic fractions of 20.97 ± 0.49 at. % Ba, 19.36 ± 0.33 at. % Ti and 59.68 ± 0.16 at. % O. These values indicate only a slight Ba enrichment and Ti depletion relative to the theoretical composition, while the oxygen content remains very close to the expected 60 at. %, in accordance with the limited BaO solubility range in BaTiO_3_. In addition, Furnace 2 shows the lowest standard deviations among the three samples, suggesting the highest compositional homogeneity. Therefore, this sample can be considered the closest to ideal BaTiO_3_ stoichiometry within the experimental uncertainty of the EDS analysis.^43–45,52^ The powder obtained in Furnace 3 shows average values of 21.48 ± 1.26 at. % Ba, 19.02 ± 0.84 at. % Ti and 59.51 ± 0.42 at. % O. These values are also reasonably close to the theoretical BaTiO_3_ composition, although they indicate a slightly larger Ba excess and Ti deficiency than those observed for Furnace 2.^43–45,52^ Moreover, the larger standard deviations, particularly for Ba and Ti, point to a greater local compositional dispersion in this sample. This behavior is consistent with the more heterogeneous microstructure and the pronounced whisker-like features observed in the SEM images, which suggest that Furnace 3 promotes stronger local variations in growth conditions even though the overall composition remains near stoichiometric.^40–42^ In contrast, the powder calcined in Furnace 1 exhibits a clearly different composition, with 24.74 ± 1.04 at. % Ba, 16.84 ± 0.69 at. % Ti and 58.42 ± 0.35 at. % O, corresponding to a Ba/Ti ratio of about 1.47 and a slightly reduced oxygen fraction. The magnitude of this deviation is substantially larger than the associated statistical scatter and clearly exceeds the expected EDS error margins, and therefore reflects a genuine compositional imbalance rather than an artifact of the analysis.^43–45,52^ This strong Ba excess is consistent with the formation of Ba-rich intermediate oxides such as BaTi_2_O_5_, as also suggested by the weak secondary reflections observed in the XRD pattern of this sample and by BaO–TiO_2_ phase-equilibria studies.^38,39,50,53^ The apparent homogeneity of the elemental maps in Figure 8 indicates that these secondary phases are finely dispersed within the BaTiO_3_ matrix and composed of the same Ba–Ti–O elements, which makes them indistinguishable in conventional EDS maps; however, their presence is captured in the bulk Ba/Ti ratio obtained from quantitative EDS analysis.^38,39,50,53^ Overall, the EDS results indicate that the powders produced in Furnaces 2 and 3 are close to stoichiometric BaTiO_3_, whereas the powder obtained in Furnace 1 remains distinctly Ba-rich and Ti-deficient.^43–45,52^ This trend agrees well with the XRD and SEM observations, confirming that Furnaces 2 and 3 promote a more complete reaction toward BaTiO_3_, while Furnace 1 favors the persistence of Ba-rich intermediate species.^40–42^ The full set of individual EDS spectra and the corresponding statistics for each element and furnace are provided in Supplementary Table S1. We emphasize that the Ba/Ti differences discussed above are not based on single-point measurements but on multiple spectra acquired from several distinct regions of each powder (Supplementary Table S1), so that the reported trend reflects the average behavior of each sample rather than local heterogeneity at an individual spot. We also note that this compositional conclusion is independently supported by the XRD results (Section 3.2), since the same Furnace-1 sample that shows the largest EDS-derived Ba excess is also the one exhibiting weak Ba-rich secondary reflections and the poorest refinement statistics; this agreement between two independent techniques strengthens confidence in the compositional trend beyond what either method could establish alone. We agree that an independent bulk technique would provide further confirmation of the Ba/Ti imbalance; we have additionally acquired XPS spectra for the Furnace-1 powder as a surface-sensitive complementary check, discussed in Section 3.6 below.

**Table 2:** Statistical Summary of SEM–EDS Quantitative Analyses (Atomic%) for Ba, Ti and O in BaTiO_3_ Powders Calcined at 1200 °C for 8 h in Furnace 1, Furnace 2 and Furnace 3^a^

Statistic	O	Ti	Ba
**Furnace 1**			
Max	58.95	17.89	26.21
Min	57.93	15.86	23.16
Average	58.42	16.84	24.74
Standard Deviation	0.35	0.69	1.04
**Furnace 2**			
Max	59.92	19.85	21.49
Min	59.5	19.01	20.23
Average	59.68	19.36	20.97
Standard Deviation	0.16	0.33	0.49
**Furnace 3**			
Max	60.19	20.39	22.47
Min	59.18	18.36	19.42
Average	59.51	19.02	21.48
Standard Deviation	0.42	0.84	1.26

a For each furnace, the maximum, minimum, average and standard deviation values are calculated from multiple spectra acquired at different regions of the powders, revealing a pronounced Ba enrichment and Ti deficiency in the Furnace-1 sample, in contrast to the near-stoichiometric compositions obtained in Furnaces 2 and 3.

**Figure 9:**
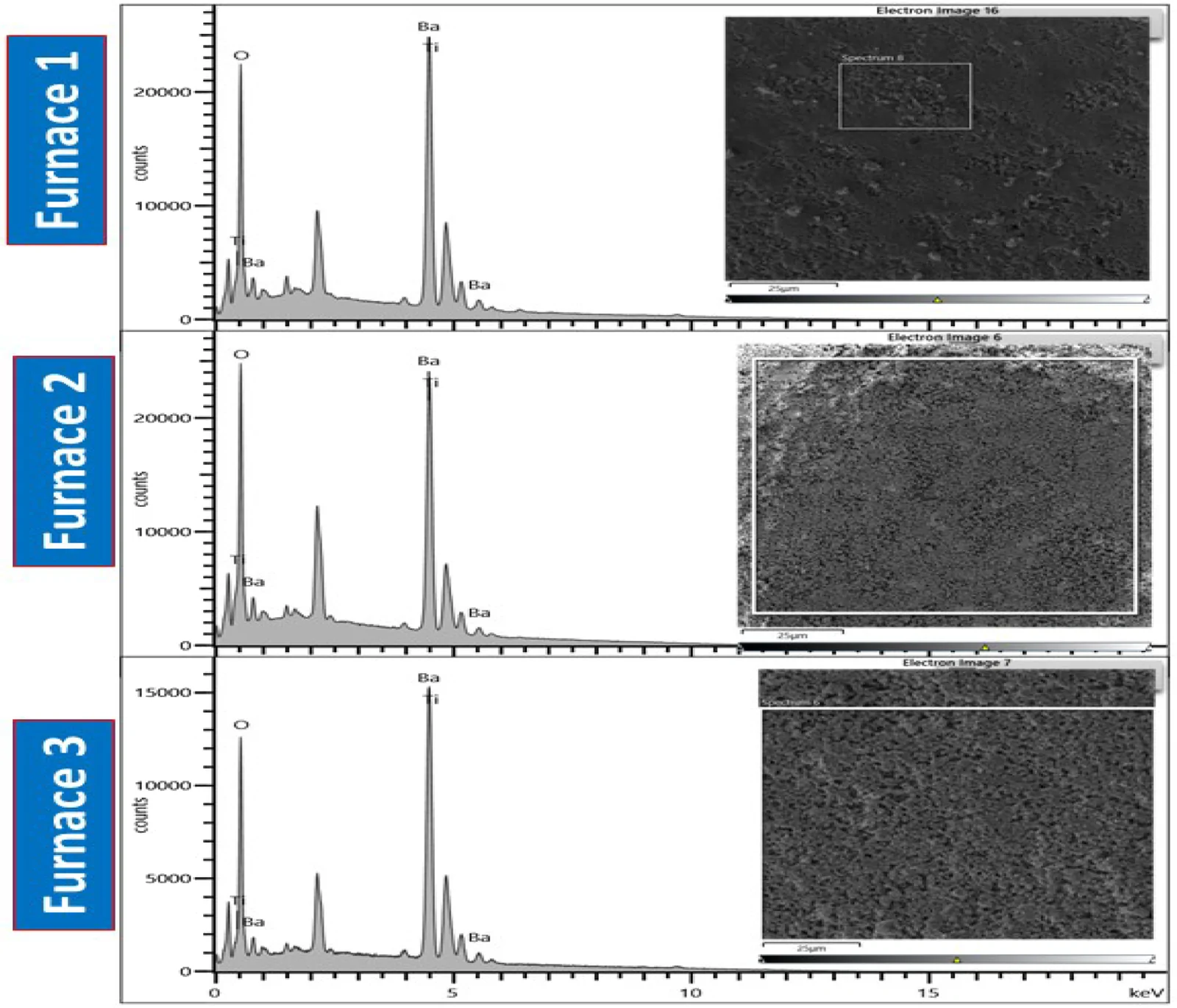
EDS spectra for BaTiO_3_ powders calcined at 1200 °C for 8 h in Furnace 1, Furnace 2 and Furnace 3. All spectra show only Ba, Ti and O peaks, confirming the absence of foreign impurity elements within the detection limits of SEM–EDS, while the relative Ba and Ti intensities reflect the small compositional differences quantified in Table 2.

The differences observed between the three BaTiO_3_ powders can be rationalized in terms of the specific thermal environments provided by the furnace systems, now directly confirmed by the three-position thermocouple measurements in Section 2 (Figure 1). The electric muffle furnace (Furnace 1) reached only ∼1028 °C at the sample position, some 170 °C below the programmed 1200 °C set point, which tends to promote Ba-rich intermediate phases (BaTi_2_O_5_), incomplete reaction and a finer, more strained microstructure, as expected from BaO–TiO_2_ phase equilibria and BaTiO_3_ formation studies.^38–42^ In contrast, the tube and Pechini-type muffle furnaces (Furnaces 2 and 3) reached and maintained a nearly isothermal ∼1190–1200 °C across the monitored positions, much closer to the programmed set point, favoring a more complete reaction toward stoichiometric BaTiO_3_ and promoting grain growth.^38–42^ Under these conditions, the Ba/Ti ratio approaches the nominal 1:1 value and the microstrain is reduced, leading to larger crystallites and, in the case of Furnace 3, to pronounced whisker-like growth driven by local supersaturation and anisotropic surface energetics.^38–42^ These results indicate that furnace-dependent factors such as chamber geometry, heating mode and temperature uniformity are critical parameters for tailoring the phase purity, grain size and morphology of BaTiO_3_ synthesized by solid-state reaction.^38–42^ Consequently, researchers aiming at fine, highly strained powders with residual intermediate phases may favor configurations similar to Furnace 1, whereas those seeking phase-pure, coarser or whisker-rich BaTiO_3_ with a more relaxed microstructure should consider furnace types and configurations closer to Furnaces 2 and 3.

### 3.6 X-ray Photoelectron Spectroscopy (XPS) Analysis

To complement the bulk-averaged EDS analysis (Section 3.5) and XRD phase analysis (Section 3.2), XPS survey and high-resolution spectra (Ba 3d, Ti 2p, O 1s, C 1s) were acquired for the calcined powders from all three furnaces, consistent with XPS being a well-established surface probe for BaTiO_3_-based ceramics and thin films.^54,55^ Survey spectra confirm only Ba, Ti, O and C are present, with no unexpected contamination (Figure 10); the core-level peaks appear at comparable binding energies and intensities across furnaces, and the C 1s signal is attributed to adventitious surface carbon, consistent with the elemental purity established by EDS (Figure 9). The fitted spectra for Furnaces 1, 2, and 3 are shown in Figures 11, 12 and 13, respectively, with binding energies, charge shifts and component fractions summarized in Table 3. Binding energies were referenced to the adventitious C 1s (C–C/C–H) component at 284.8 eV, giving charge shifts of +1.20 eV (Furnace 1), + 1.34 eV (Furnace 2) and +1.05 eV (Furnace 3), consistent with modest sample charging typical of a poorly conducting oxide powder. After Shirley background subtraction, the C 1s spectra resolved into three components: C–C/C–H (284.8 eV), C–O/C–OH (285.7–285.8 eV), and a smaller carbonate-like O–C=O component (288.8–289.0 eV) consistent with surface-adsorbed CO_2_/carbonate, in agreement with previous reports of residual surface carbon on BaTiO_3_ ceramics.^56^ This component accounted for 9.1%, 16.9% and 10.2% of the C 1s area in Furnaces 1, 2 and 3, respectively. The O 1s envelope resolved into a lattice-oxygen component (528.8–529.0 eV) and a broader surface hydroxyl/carbonate component (530.7–531.1 eV), the latter dominating the O 1s area (70–72%) in all samples. The Ti 2p spectra showed a single Ti^4+^ doublet (2p3/2 at 457.8–458.0 eV, 2p1/2 at 463.5–463.7 eV, splitting ≈5.7 eV) with no Ti^3+^ component the latter would be expected to appear as a distinct lower-binding-energy shoulder near 456–457 eV in partially reduced BaTiO_3_.^57^ The Ba 3d spectra showed a single Ba^2+^ doublet (3d5/2 at 778.7–779.2 eV, 3d3/2 at 794.0–794.5 eV, splitting ≈15.3–15.4 eV) with no distinct BaCO_3_-like component, consistent with binding-energy assignments reported for related B-site-modified BaTiO_3_ systems.^54,55^ These chemical-state assignments are indistinguishable between furnaces, despite the compositional and phase differences indicated by EDS and XRD. We do not report XPS-derived Ba:Ti:O atomic percentages, as this requires sensitivity factors and a transmission function calibrated for this specific spectrometer, which were not available to us. The XPS results are therefore presented as qualitative, chemical-state-level evidence confirming a consistent Ti^4+^/Ba^2+^/lattice-oxide environment across all three furnaces rather than as an independent quantitative confirmation of the EDS/XRD-based Ba/Ti trend.

**Figure 10:**
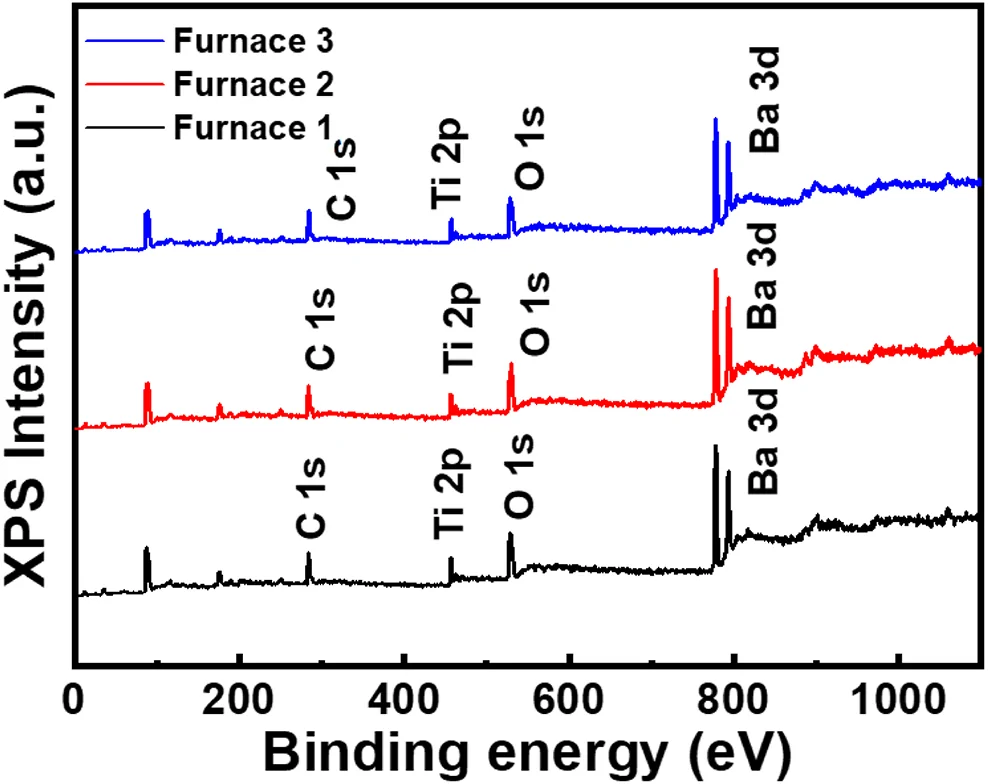
XPS survey spectra of BaTiO_3_ powders calcined in Furnace 1, Furnace 2, and Furnace 3, showing the presence of Ba, Ti, O, and C with no detectable elemental impurities.

**Table 3:** Charge-Referencing Shifts and Key XPS Binding Energies (Ba 3d5/2, Ti 2p3/2, O 1s Lattice Component) and Carbonate-Type C 1s Area Fraction for BaTiO_3_ Powders Calcined at 1200 °C for 8 h in Furnace 1, Furnace 2 and Furnace 3

Furnace	Charge shift (eV)	Ba 3d5/2 (eV)	Ti 2p3/2 (eV)	O 1s Lattice (eV)	Carbonate C 1s (%)
1	+1.20	778.97	457.96	529.03	9.1
2	+1.34	779.16	457.94	528.99	16.9
3	+1.05	778.66	457.77	528.83	10.2

**Figure 11:**
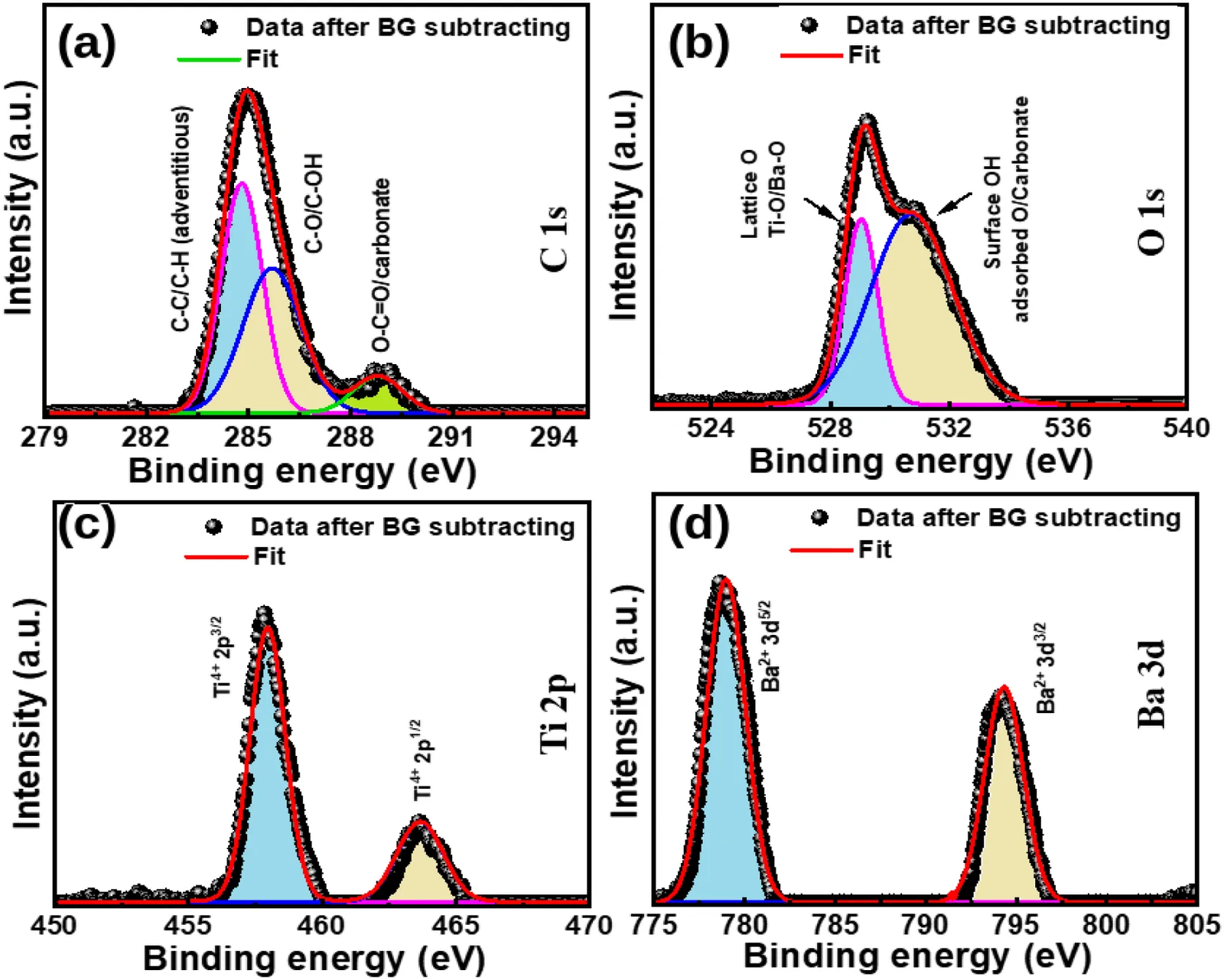
X-ray photoelectron spectra (charge-referenced, Shirley-background-subtracted) of the BaTiO_3_ powder calcined in Furnace 1: (a) C 1s, (b) O 1s, (c) Ti 2p and (d) Ba 3d regions, with pseudo-Voigt component fits (see Table 3 for fitted binding energies, widths and area fractions).

**Figure 12:**
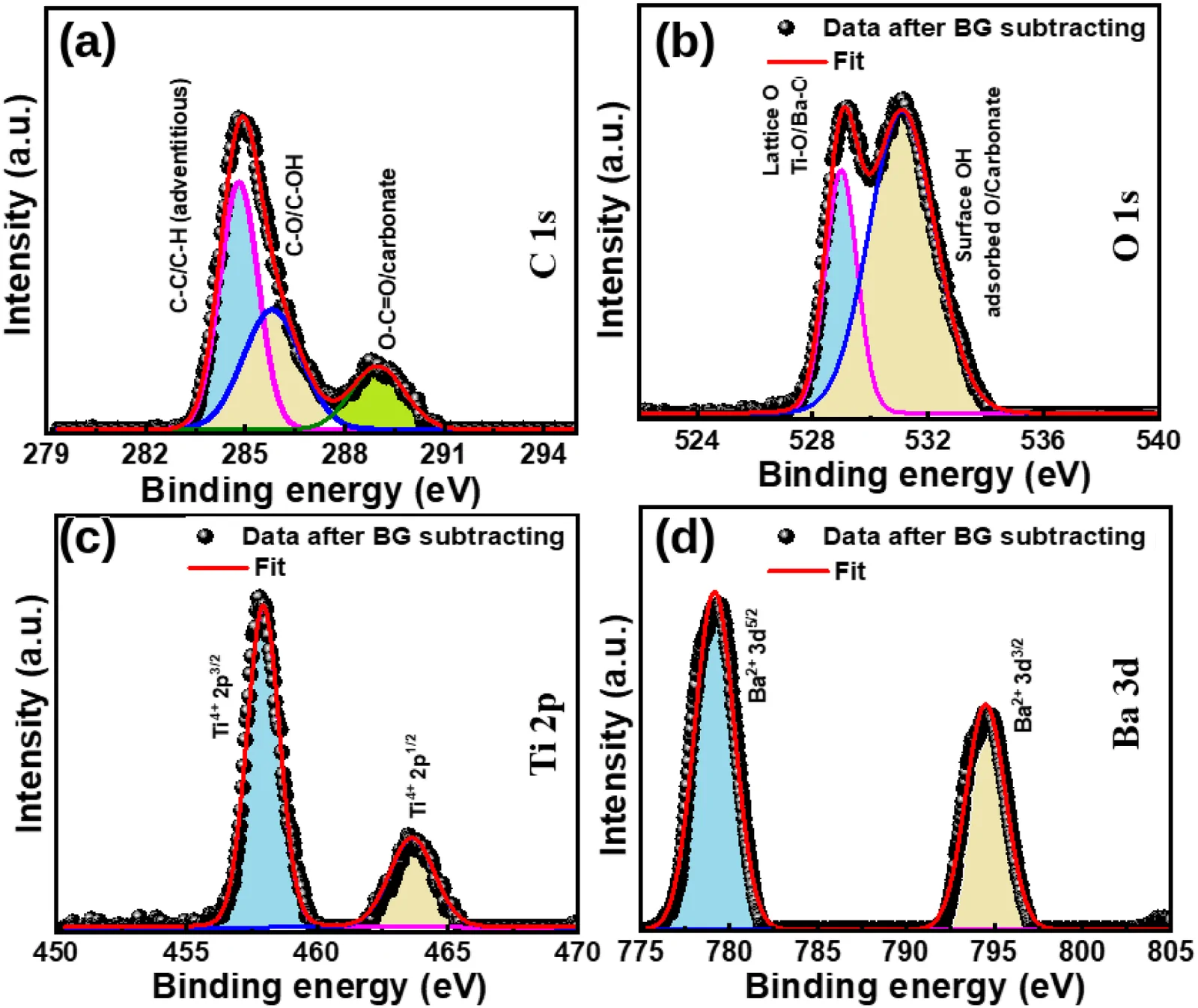
X-ray photoelectron spectra of the BaTiO_3_ powder calcined in Furnace 2: (a) C 1s, (b) O 1s, (c) Ti 2p and (d) Ba 3d regions, processed and fitted as in Figure 11.

**Figure 13:**
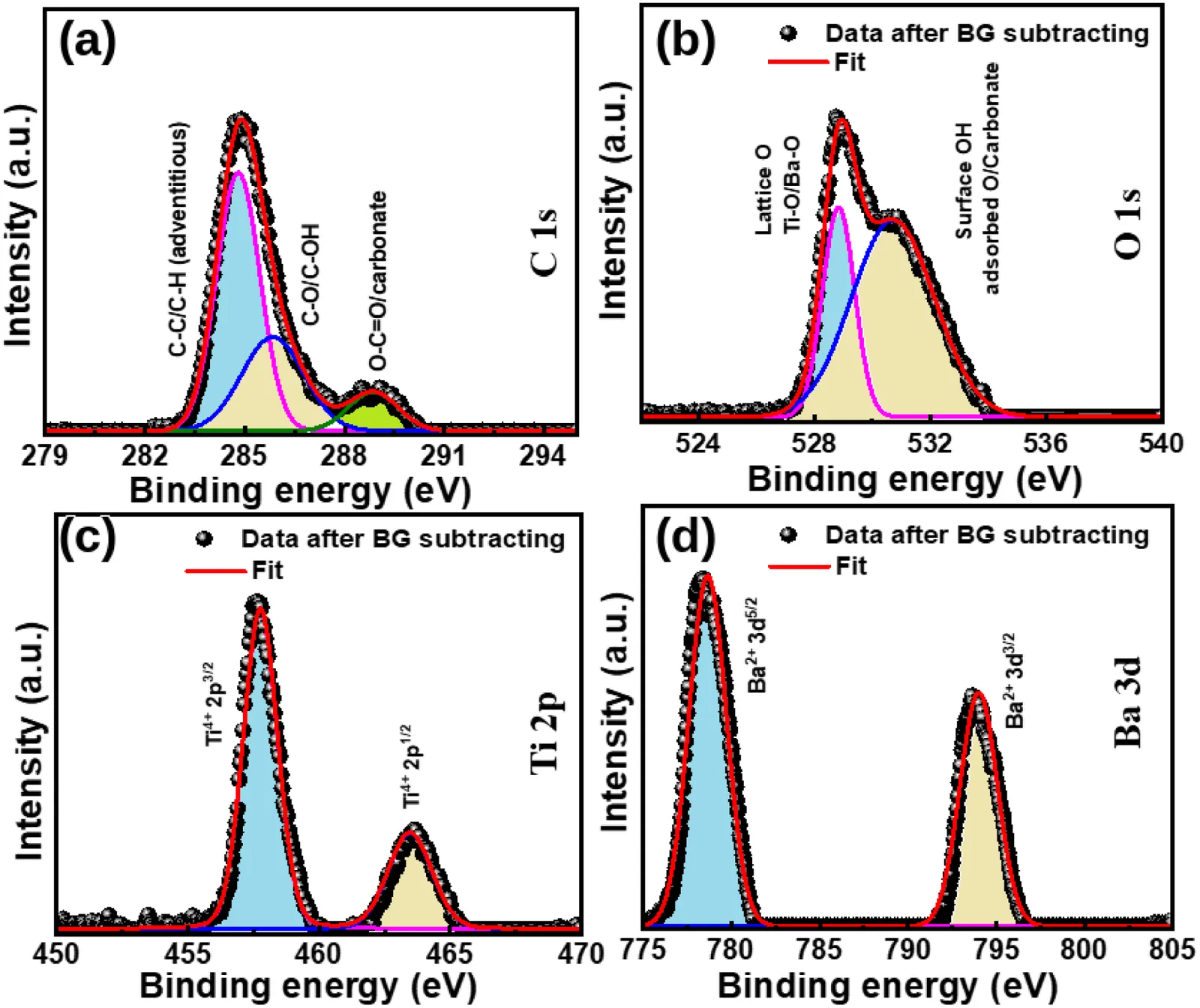
X-ray photoelectron spectra of the BaTiO_3_ powder calcined in Furnace 3: (a) C 1s, (b) O 1s, (c) Ti 2p and (d) Ba 3d regions, processed and fitted as in Figure 11.

## 4 Conclusion

This study shows that BaTiO_3_ powders produced from an identical ball-milled precursor batch and calcined under the same nominal conditions can exhibit significantly different structural and microstructural properties when treated in different furnaces. All powders were predominantly tetragonal BaTiO_3_; however, the sample produced in Furnace 1 contained a substantial Ba-rich BaTi_2_O_5_ secondary phase (82 wt % BaTiO_3_/18 wt % BaTi_2_O_5_, from two-phase Rietveld refinement), poorer refinement quality, smaller crystallite sizes and higher microstrain than the powders treated in Furnaces 2 and 3. SEM and quantitative EDS confirmed this distinction, indicating a finer and more Ba-rich product from Furnace 1, whereas Furnaces 2 and 3 generated coarser, nearly stoichiometric powders. Direct three-position thermocouple measurements identified the origin of this behavior: Furnace 1 reached only ∼1028 °C at the sample position, roughly 170 °C below the nominal 1200 °C target, while Furnaces 2 and 3 remained close to isothermal at the intended set point. The results do not independently determine a single physical origin for the residual Furnace-2-versus-Furnace-3 morphological differences; nonetheless, they clearly demonstrate that furnace identity acts as a hidden experimental variable in the calcination of BaTiO_3_. Thus, furnace identity must be controlled, documented and considered when developing reproducible synthesis protocols and when comparing ceramic processing outcomes across laboratories. We therefore recommend that furnace identity and basic furnace characteristics (type, geometry and atmosphere) be reported explicitly in ceramic synthesis studies to improve interlaboratory reproducibility. We reiterate that these conclusions are based on a single calcination run per furnace and should be understood as demonstrating that furnace-dependent effects of this magnitude are possible, rather than establishing their general statistical prevalence; replicate runs per furnace and an independent bulk-composition technique (e.g., ICP-OES) represent natural next steps to build on the present findings.

## Supplementary Material



## Data Availability

All data are confidential and are included in the manuscript and the Supporting Information file.

## Notes

Supplementary SEM images (Figures S1–S3), EDS elemental maps (Figures S4–S6), representative EDS spectra (Figures S7–S9), and full SEM–EDS quantitative results with statistical summary (Table S1) for BaTiO_3_ powders calcined in Furnaces 1, 2, and 3; position-resolved temperature and XRD verification for the Furnace-1 sample are provided in Figure S10 (PDF)
