# Supplementary material for: Furnace-Dependent Phase Evolution of Ball-Milled BaTiO3 Powders under Nominally Identical Calcination Conditions
Source: ACS Omega. 2026 Sep 23;11(39):59210–25. doi: 10.1021/acsomega.6c04998 (PMC13648061; doi:10.1021/acsomega.6c04998)
Supplement: Supplementary file 1 [file ao6c04998_si_001.pdf]

Supplemental information

Furnace-Dependent Phase Evolution of Ball-Milled $BaTiO_3$ Powders under Nominally Identical Calcination Conditions

Mahmoud S. Alkathy[1*]. Person Pereira Neves[1*]and, Aníbal Thiago Bezerra[1], Gabriel Monteiro A. da Rocha[1], Leandro Sanches[1], Jose Antonio Eiras[2]

1. Physics Department, Institute of Exact Sciences- UNIFAL-MG, Alfenas - MG, 37133-840, Brazil
2. Physics Department, Institute of Exact Sciences-UFSCar- São Carlos, SP, 13565-905, Brazil

*Corresponding Author

mahmoud.kathy@unifal-mg.edu.br

person.neves@unifal-mg.edu.br

Table S1. SEM–EDS quantitative results (atomic%) for selected spectra acquired from $BaTiO_3$ powders calcined at 1200 °C for 8 h in Furnace 1, Furnace 2 and Furnace 3. The spectra from Furnace 1 correspond to Ba-rich intermediate domains embedded in the $BaTiO_3$ matrix, whereas the spectra from Furnaces 2 and 3 are representative of nearly stoichiometric $BaTiO_3$ regions, consistent with the average compositions summarized in Table 2 and the Ba-rich secondary features identified by XRD for Furnace 1.

**Furnace 1**						
	Result Type			Atomic%		
Label	Spectrum 17	Spectrum 18	Spectrum 19	Spectrum 20	Spectrum 21	Spectrum 8
O	58.23	57.93	58.95	58.39	58.63	58.38
Ti	16.47	15.86	17.89	16.79	17.26	16.77
Ba	25.3	26.21	23.16	24.82	24.12	24.85
Total	100	100	100	100	100	100
**Furnace 2**						
	Result Type			Atomic%		
Label	Spectrum 23	Spectrum 24	Spectrum 25	Spectrum 26	Spectrum 27	Spectrum 7
O	59.64	59.83	59.5	59.56	59.92	59.61
Ti	19.29	19.66	19.01	19.12	19.85	19.22
Ba	21.07	20.51	21.49	21.32	20.23	21.17
Total	100	100	100	100	100	100
**Furnace 3**						
	Result Type			Atomic%		
Label	Spectrum 36	Spectrum 37	Spectrum 38	Spectrum 39	Spectrum 11	Label
O	59.18	59.2	59.6	60.19	59.38	O
Ti	18.36	18.39	19.19	20.39	18.75	Ti
Ba	22.47	22.41	21.21	19.42	21.87	Ba
Total	100	100	100	100	100	Total

Supplemental information

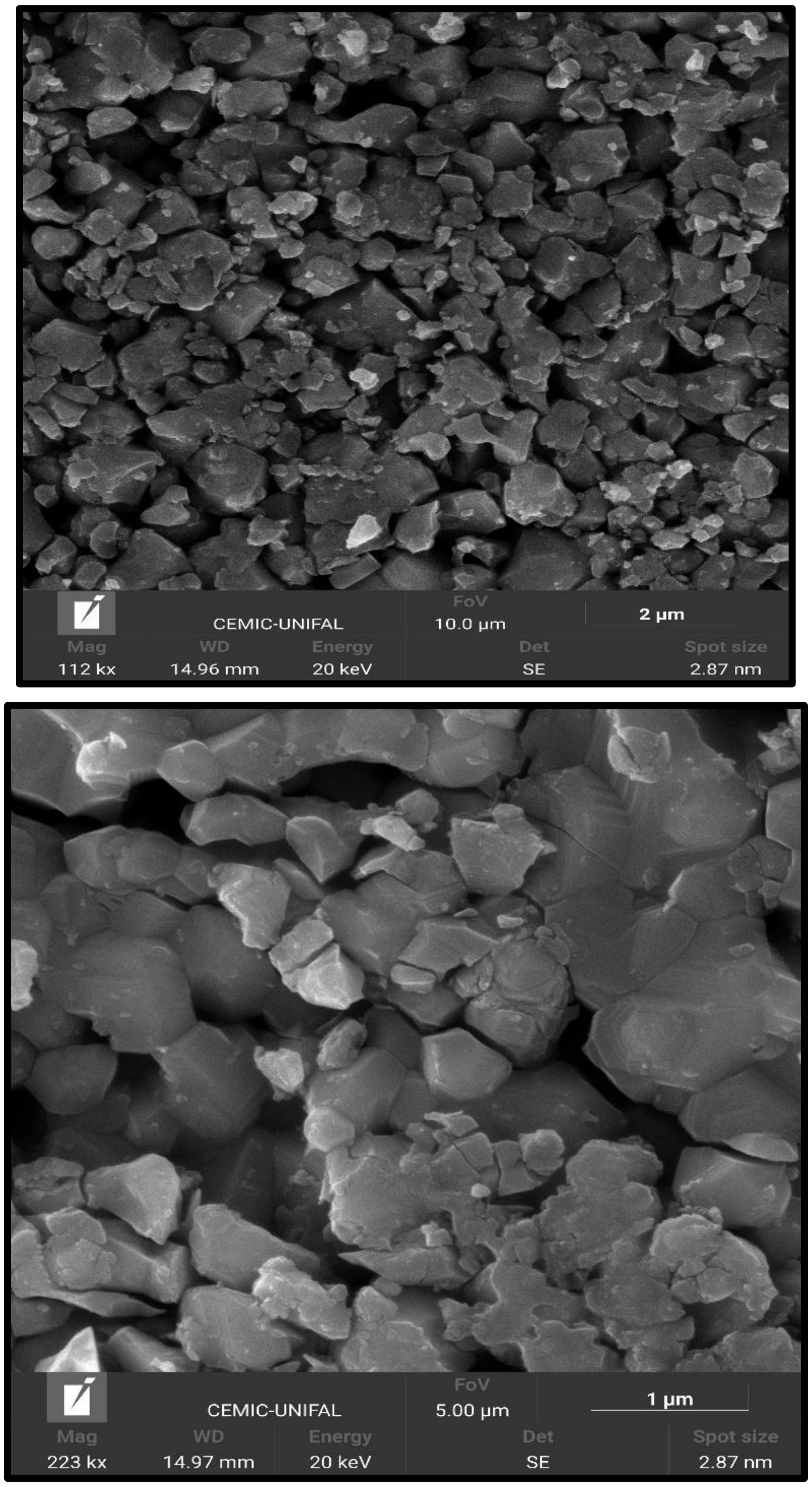

Figure S1. SEM micrographs of $BaTiO_3$ powders calcined at 1200 °C for 8 h in Furnace 1 measured at two different magnifications.

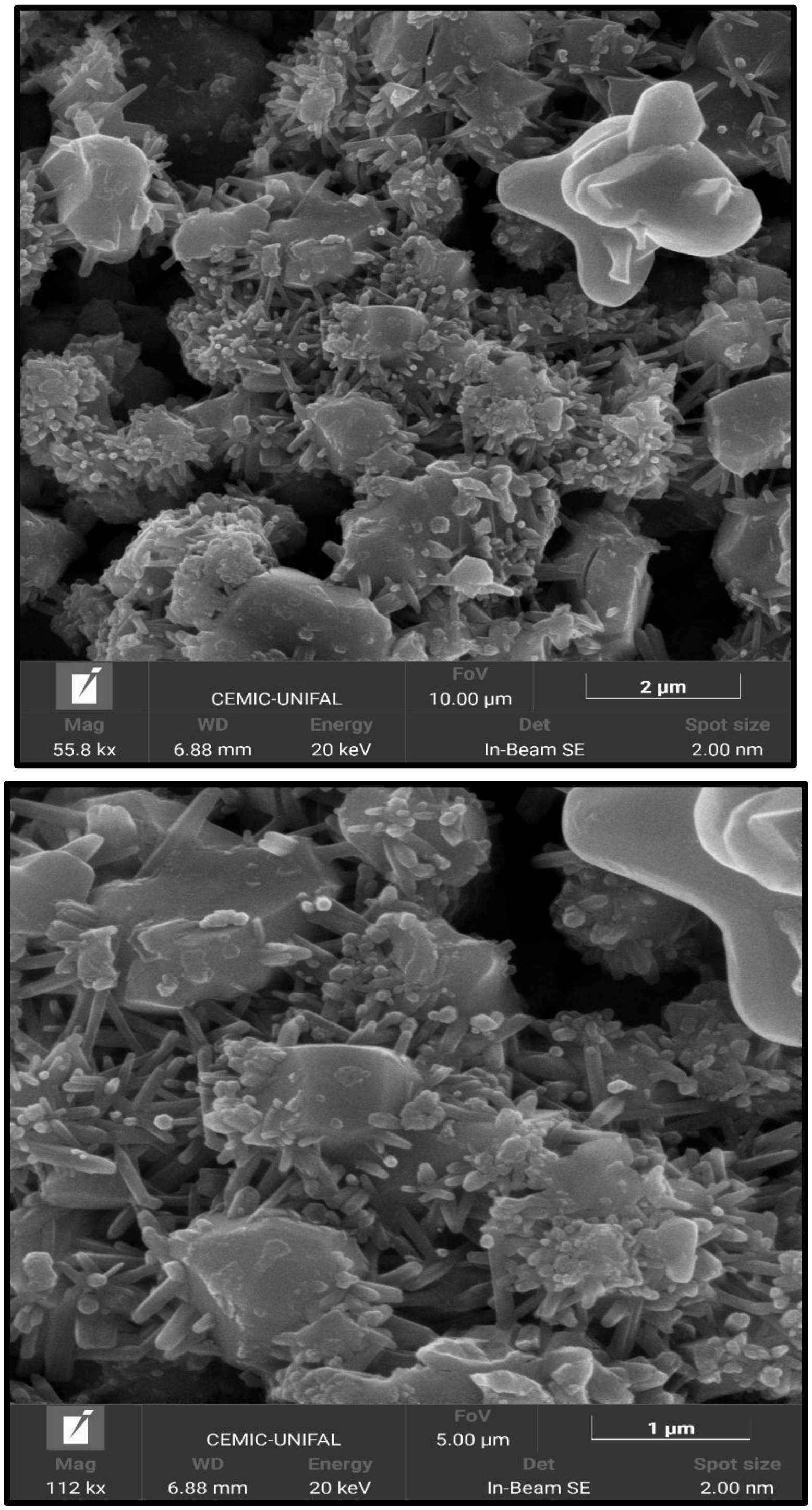

Figure S2. SEM micrographs of $BaTiO_3$ powders calcined at 1200 °C for 8 h in Furnace 2 measured at two different magnifications.

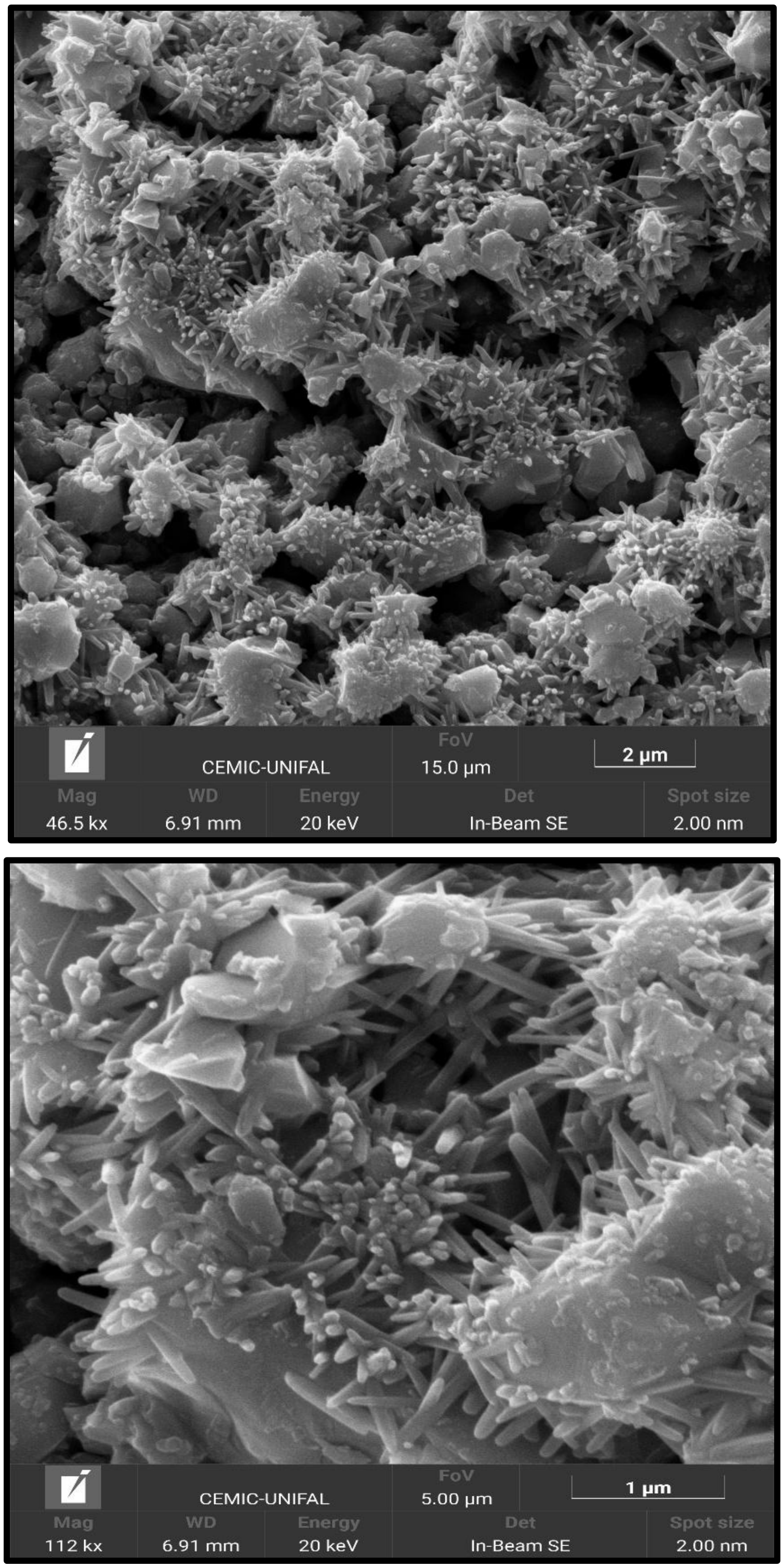

Figure S3. SEM micrographs of $BaTiO_3$ powders calcined at 1200 °C for 8 h in Furnace 3 measured at two different magnifications

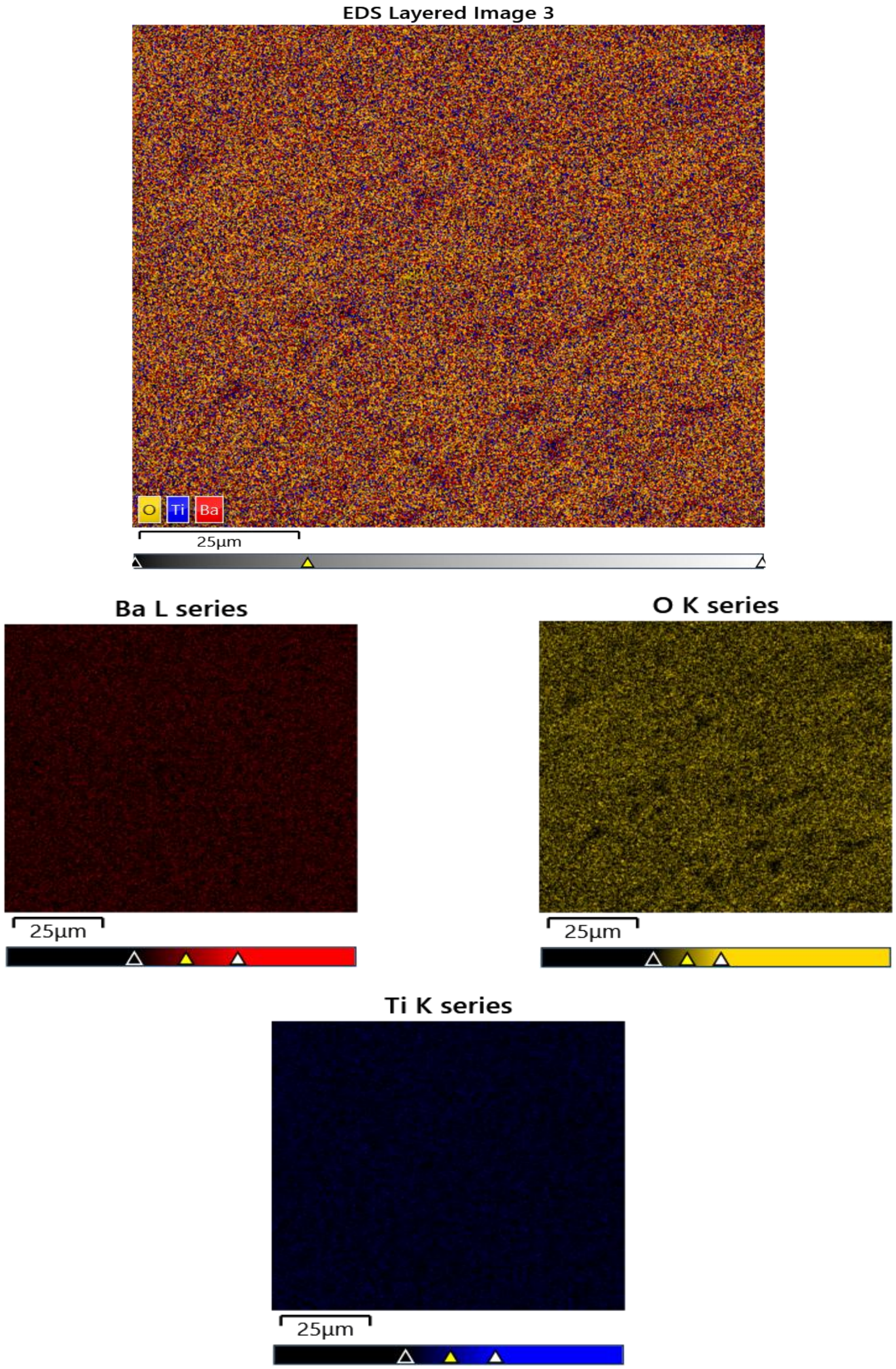

Figure S4. EDS elemental maps of Ba (Ba L series), O (O K series) and Ti (Ti K series) for $BaTiO_3$ powders calcined at 1200 °C for 8 h in Furnace 1.

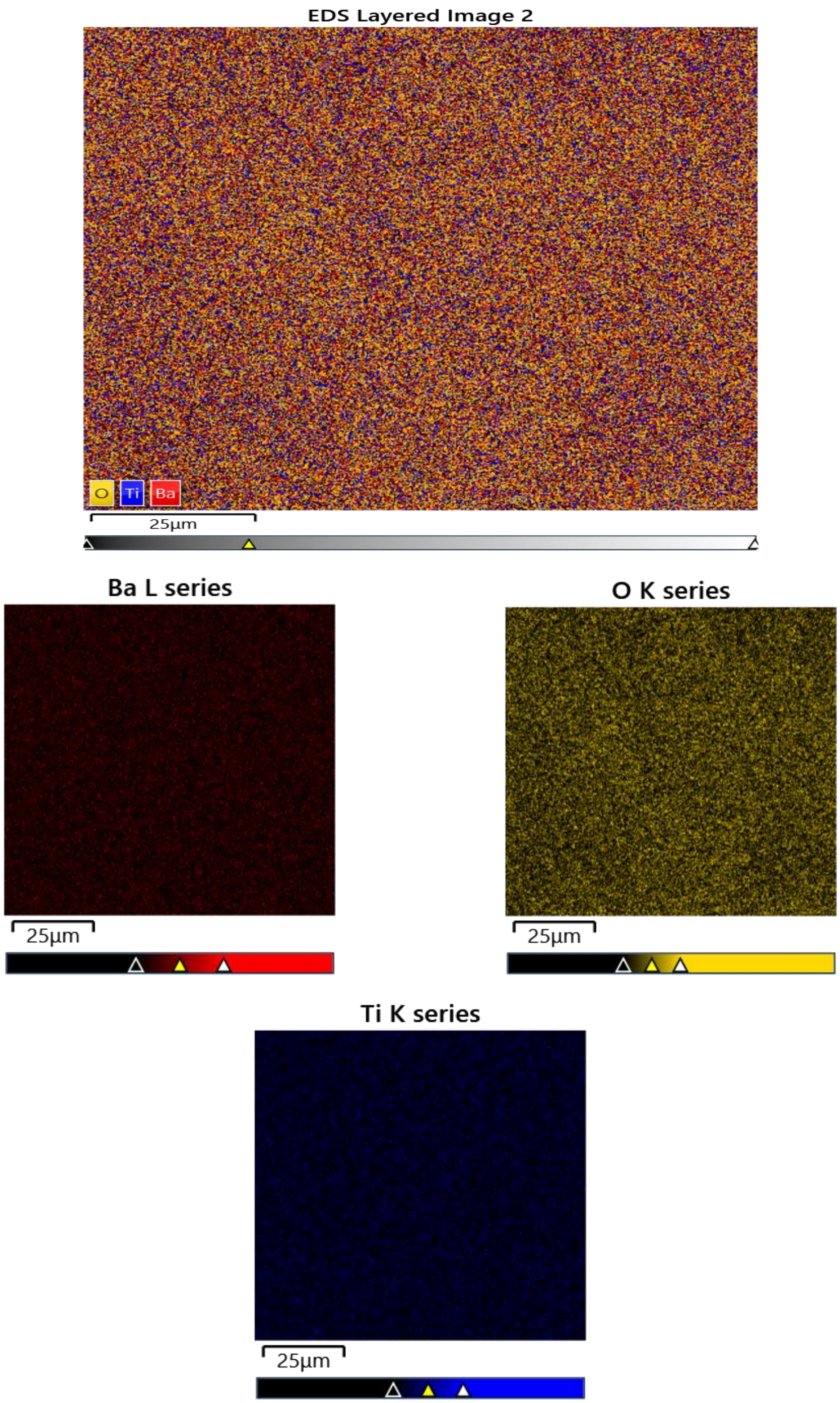

Figure S5. EDS elemental maps of Ba (Ba L series), O (O K series) and Ti (Ti K series) for $BaTiO_3$ powders calcined at 1200 °C for 8 h in Furnace 2.

Supplemental information

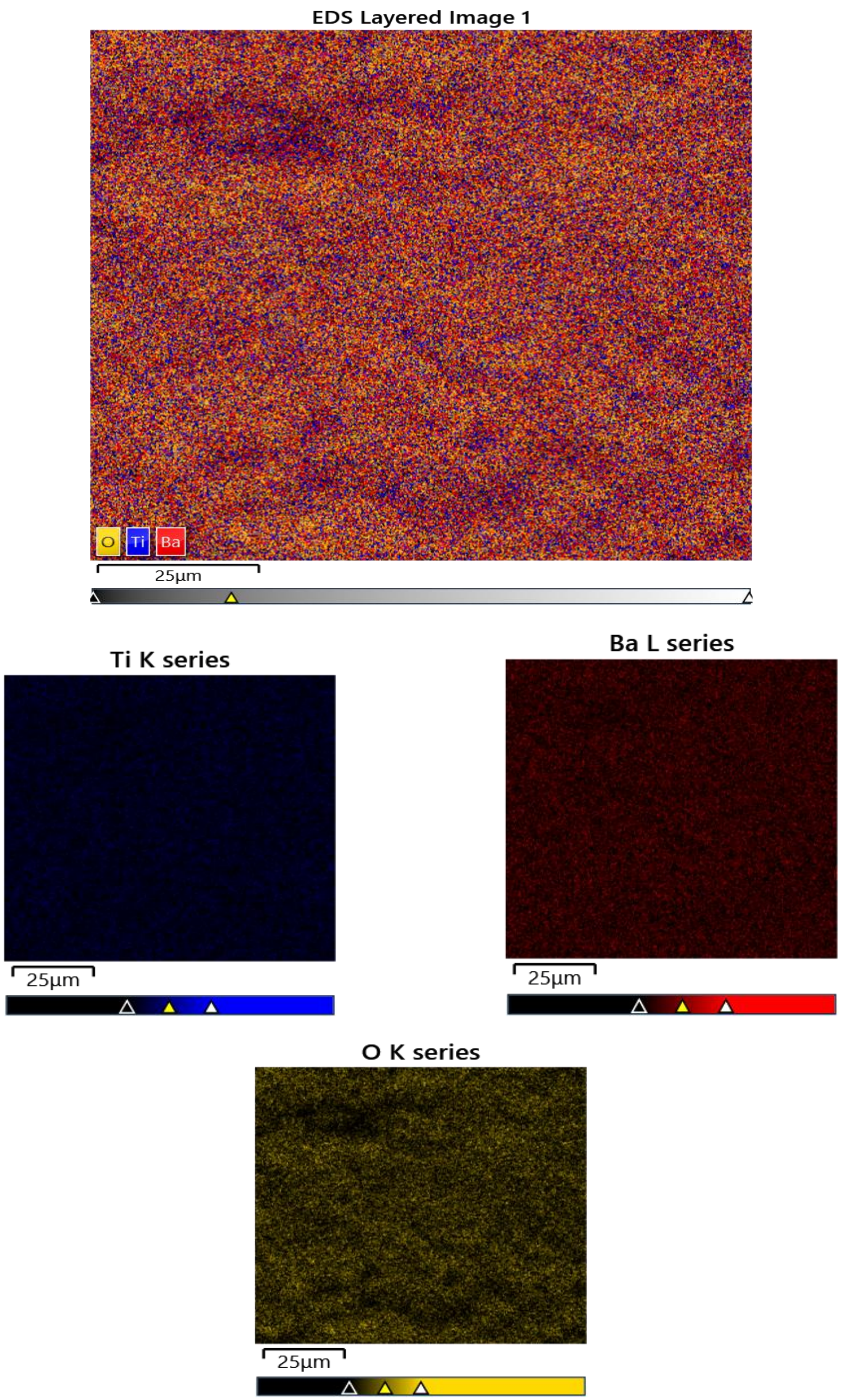

Figure S6. EDS elemental maps of Ba (Ba L series), O (O K series) and Ti (Ti K series) for $BaTiO_3$ powders calcined at 1200 °C for 8 h in Furnace 3.

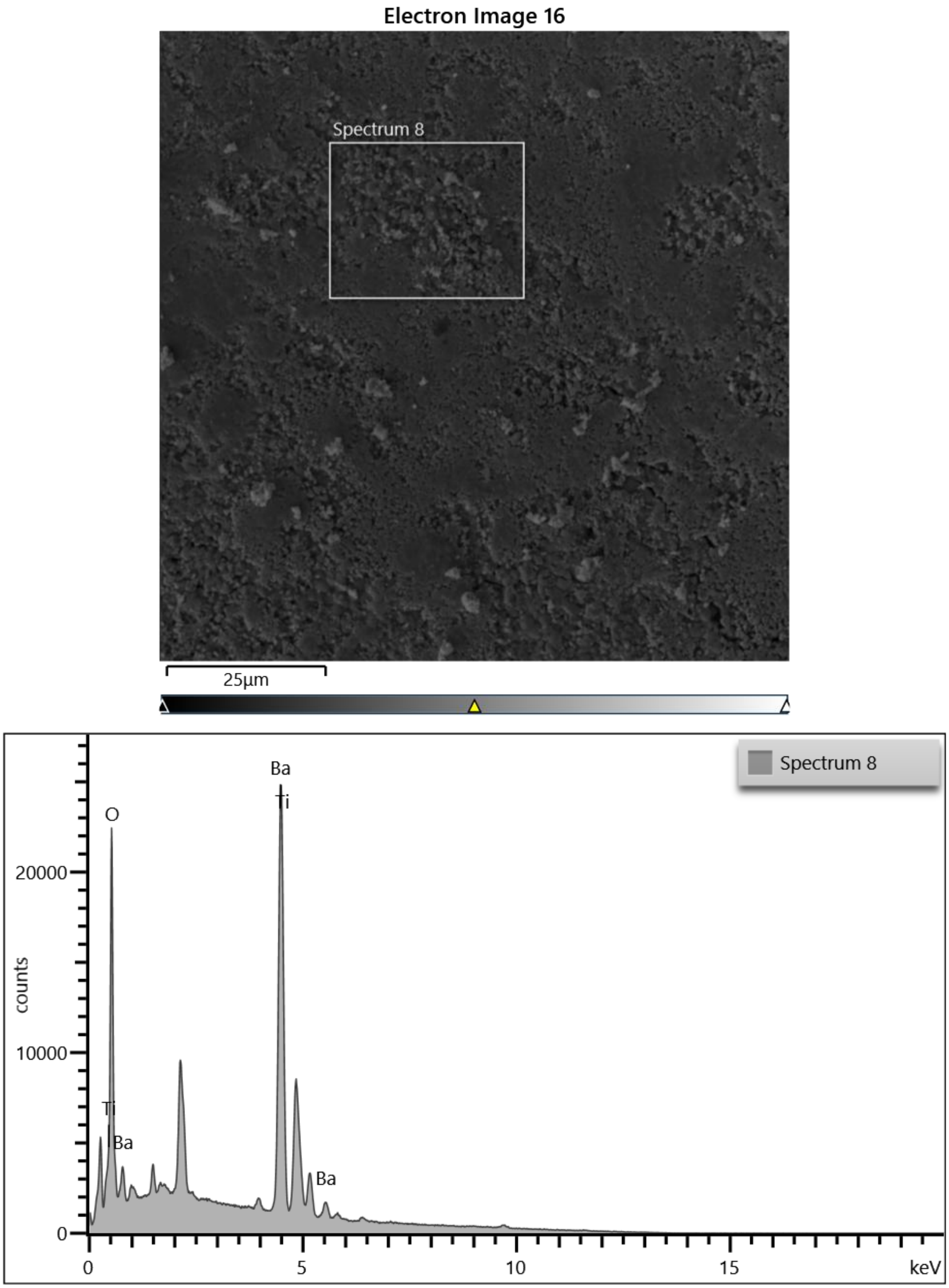

Figure S7. EDS spectra for $BaTiO_3$ powders calcined at 1200 °C for 8 h in Furnace 1.

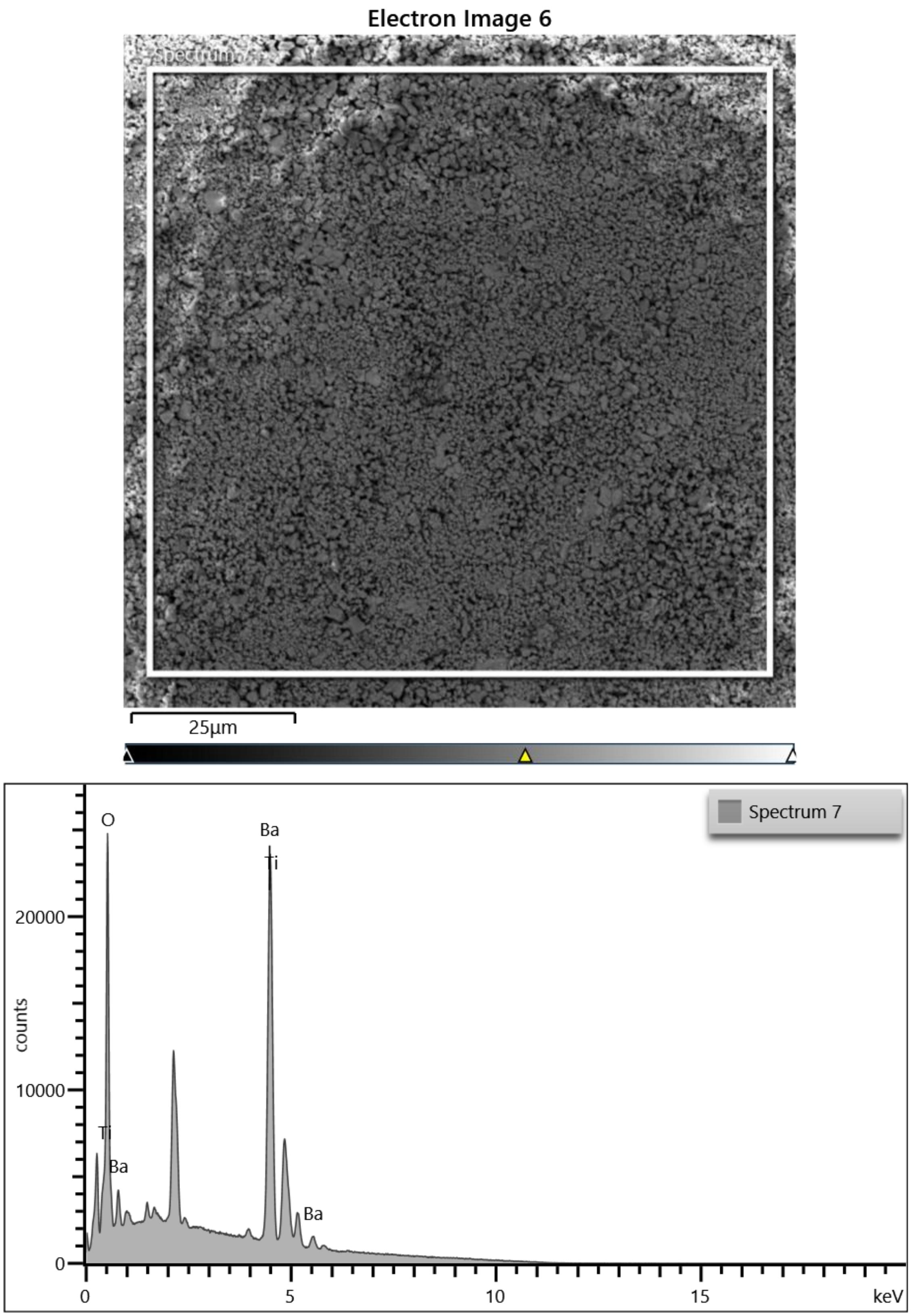

Figure S8. EDS spectra for $BaTiO_3$ powders calcined at 1200 °C for 8 h in Furnace 2.

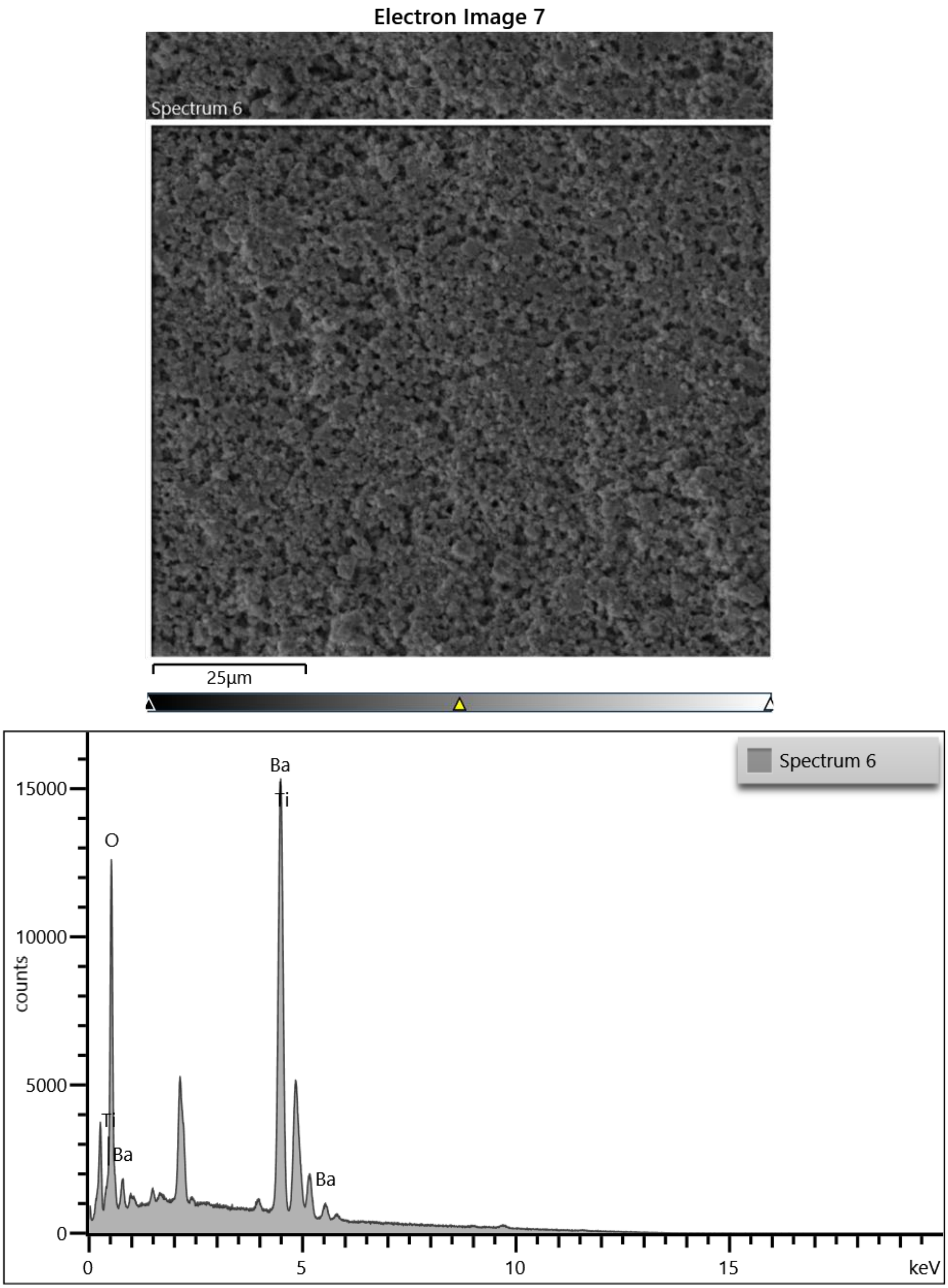

Figure S9. EDS spectra for $BaTiO_3$ powders calcined at 1200 °C for 8 h in Furnace 3.

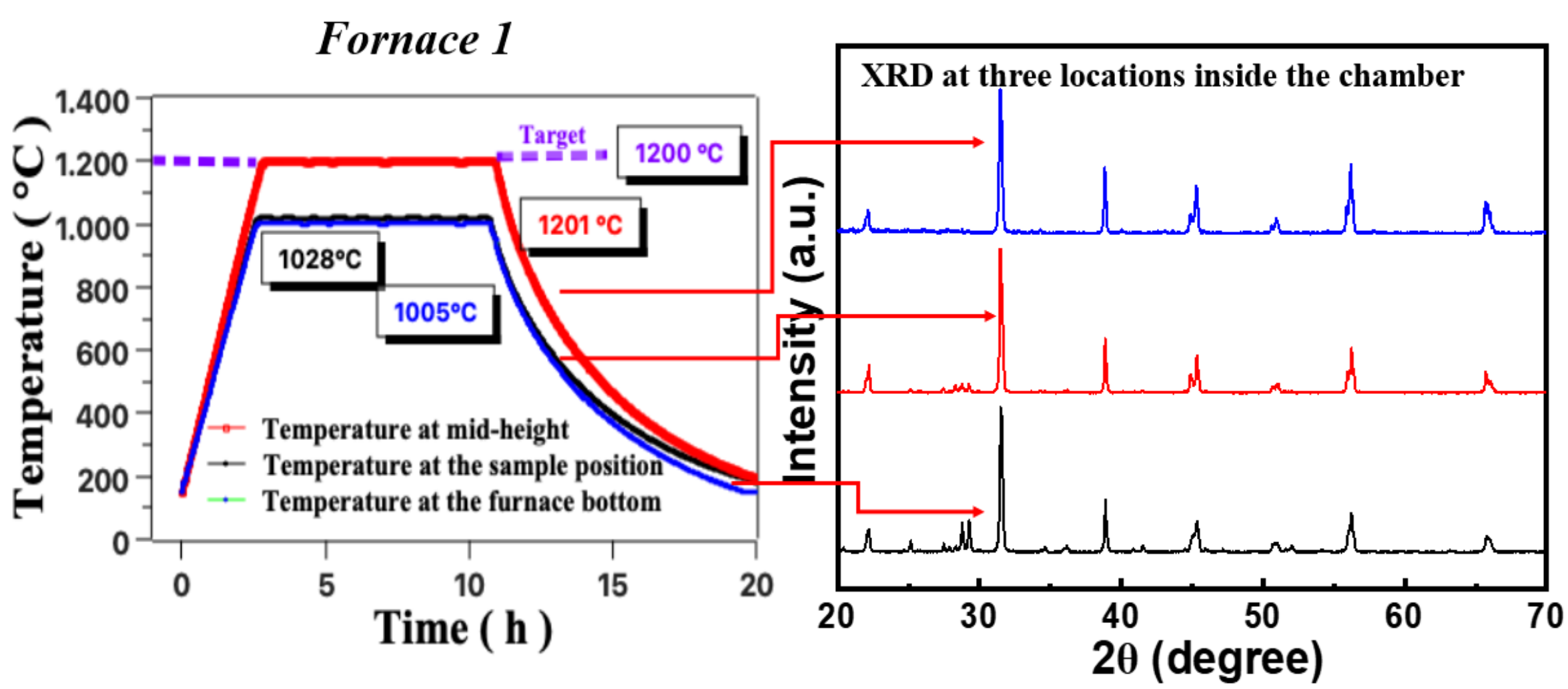

Figure S10. Position-resolved verification for the Furnace-1 sample. Left: temperature profile measured by three K-type thermocouples (mid-height, sample position, furnace bottom) during the programmed 1200 °C / 8 h calcination run (reproduced from Figure 1 of the main text). Right: XRD patterns of powder collected from the three corresponding chamber locations mid-height (blue, 1201 °C), sample position (red, 1028 °C) and furnace bottom (black, 1005 °C), as indicated by the connecting arrows. The pattern from the coolest zone (furnace bottom) shows additional low-angle reflections not present in the hottest zone (mid-height), consistent with a more complete reaction toward phase-pure $BaTiO_3$ at higher local temperature and a greater degree of residual, incompletely reacted Ba-rich phase in the cooler regions of the chamber. This position-resolved comparison, obtained within a single calcination run, provides direct physical evidence that the furnace-1-specific outcome tracks local temperature rather than run-to-run variability alone; it does not replace the replicate full calcination runs that would be needed to quantify furnace-to-furnace run variability, which remains a limitation of the present study.
